# Muscle Injuries Induce a Prostacyclin‐PPAR*γ*/PGC1a‐FAO Spike That Boosts Regeneration

**DOI:** 10.1002/advs.202301519

**Published:** 2023-05-04

**Authors:** Lanfang Luo, Yan‐Jiang Benjamin Chua, Taoyan Liu, Kun Liang, Min‐Wen Jason Chua, Wenwu Ma, Jun‐Wei Goh, Yuefan Wang, Jiali Su, Ying Swan Ho, Chun‐Wei Li, Ke Hui Liu, Bin Tean Teh, Kang Yu, Ng Shyh‐Chang

**Affiliations:** ^1^ Institute of Zoology Chinese Academy of Sciences Beijing 100101 P. R. China; ^2^ Beijing Institute for Stem Cell and Regenerative Medicine Institute for Stem Cell and Regeneration Chinese Academy of Sciences Beijing 100101 P. R. China; ^3^ University of Chinese Academy of Sciences Beijing 100049 P. R. China; ^4^ NUS Graduate School for Integrative Sciences and Engineering National University of Singapore Singapore City 119077 Singapore; ^5^ Genome Institute of Singapore Institute of Molecular and Cell Biology Agency for Science Technology and Research Singapore City 138672 Singapore; ^6^ Bioprocessing Technology Institute Agency for Science Technology and Research Singapore City 138668 Singapore; ^7^ Department of Clinical Nutrition Peking Union Medical College Hospital Chinese Academy of Medical Sciences & Peking Union Medical College Beijing 100730 P. R. China; ^8^ Laboratory of Cancer Therapeutics Program in Cancer and Stem Cell Biology Duke‐NUS Medical School Singapore City 169857 Singapore; ^9^ Division of Medical Science Laboratory of Cancer Epigenome National Cancer Centre Singapore Singapore City 119074 Singapore

**Keywords:** MSI, muscle regeneration, muscle stem cell (MuSC), myobolites, prostanoids

## Abstract

It is well‐known that muscle regeneration declines with aging, and aged muscles undergo degenerative atrophy or sarcopenia. While exercise and acute injury are both known to induce muscle regeneration, the molecular signals that help trigger muscle regeneration have remained unclear. Here, mass spectrometry imaging (MSI) is used to show that injured muscles induce a specific subset of prostanoids during regeneration, including PGG1, PGD2, and the prostacyclin PGI2. The spike in prostacyclin promotes skeletal muscle regeneration via myoblasts, and declines with aging. Mechanistically, the prostacyclin spike promotes a spike in PPAR*γ*/PGC1a signaling, which induces a spike in fatty acid oxidation (FAO) to control myogenesis. LC–MS/MS and MSI further confirm that an early FAO spike is associated with normal regeneration, but muscle FAO became dysregulated during aging. Functional experiments demonstrate that the prostacyclin‐PPAR*γ*/PGC1a‐FAO spike is necessary and sufficient to promote both young and aged muscle regeneration, and that prostacyclin can synergize with PPAR*γ*/PGC1a‐FAO signaling to restore aged muscles’ regeneration and physical function. Given that the post‐injury prostacyclin‐PPAR*γ*‐FAO spike can be modulated pharmacologically and via post‐exercise nutrition, this work has implications for how prostacyclin‐PPAR*γ*‐FAO might be fine‐tuned to promote regeneration and treat muscle diseases of aging.

## Graphical abstract

Using mass spectrometry imaging, several prostaglandin lipids and fatty acid oxidation (FAO) intermediates are discovered to be injury‐induced myobolites. Detailed studies in vitro and in vivo reveal that the prostacyclin PGI2‐PPAR*γ*/PGC1a‐FAO axis is a key myobolite pathway that spikes transiently to boost muscle regeneration in young mammals, but declines during aging. These myobolites can be targeted to treat aged muscles.

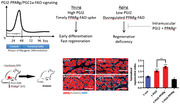

## 1 Introduction

Skeletal muscles are a well‐established model system for studying tissue regeneration.^[^ ^1^ ^]^ In response to injury, Pax7+ muscle stem cells (MuSCs) are activated by molecular signals to enter a proliferative state.^[^ ^2^ ^]^ Such activated muscle stem cells or myoblasts are regulated by the muscle‐specific transcription factor MyoD. Upon commitment, myoblasts begin to express myogenin (MYOG) and start differentiating into non‐proliferative myocytes. MYOG+ myocytes are fusion‐competent and subsequently fuse into multi‐nucleated myotubes and myofibers with diameter ≈100 µm, and which express high levels of myosin and sarcomeric *α*‐actinin. Due to the complexity of molecular changes that occur during the transition from cellular proliferation to differentiation, the metabolic signals and requirements of these states are likely to be complex and dynamic as well. Hence the metabolic changes that promote cell fate transitions in myoblast differentiation had remained unclear. It was also unclear how these metabolic transitions are dysregulated during aging, when muscles undergo degenerative atrophy or sarcopenia, or if they could be reversed to restore regenerative self‐renewal in aged muscles.^[^ ^3^ ^]^

Intracellular metabolism in bulk muscle has been thoroughly studied for over a century. In contrast, extracellular secretion of metabolic signals by specific muscle cell types and their effects on local stem cell compartments, especially after acute injury or physical exercise, has only become clearer recently. Myobolites are a new class of metabolites, induced by muscle injury or exercise activity, which might exert beneficial effects on muscular and overall health.^[^ ^4^ ^]^ While several myokines, such as FGF21 and irisin, and inflammatory cytokines^[^ ^5^ ^]^ have been found to partially explain the health benefits of physical exercise, it has remained unclear whether metabolites produced in skeletal muscles could also promote muscle function and health. Previous studies had uncovered a role for a few prostaglandins in regulating myoblast motility and fusion in vitro^[^ ^6^ ^]^ and especially prostaglandin E2 (PGE2) in MuSC proliferation^[^ ^7^ ^]^, but it was unclear if there exist other myobolites that could directly induce myoblast differentiation in vivo, the key event for new myofiber formation during muscle regeneration.

One bottleneck in the myobolite field has been the lack of omics technologies to screen, identify and quantify low concentrations of metabolites in situ, to find new myobolites localized in injured regenerative regions vs. normal adjacent regions‐of‐interest (ROI). In recent years, spatial metabolomics and especially mass spectrometry imaging (MSI) have emerged as a possible technology with the requisite spatial accuracy, mass resolution, and sensitivity to achieve this goal.^[^ ^8^ ^]^ MSI can easily separate one ROI from another ROI for mass spectrometry analysis. Here, we used matrix‐assisted laser desorption ionization (MALDI)‐based MSI to reveal that prostacyclin is a myobolite, and that the injury‐induced prostacyclin‐PPAR*γ*/PGC1a‐FAO spike promotes myoblast proliferation, differentiation, and muscle regeneration after injury. We further revealed that in aged muscles, there is a decrease in prostacyclin and a delayed spike in FAO, and reversal of these defects can restore muscle regeneration and contractile function in sarcopenia. These findings bear important implications for our understanding of how the prostacyclin‐PPAR*γ*/PGC1a‐FAO axis is spatio‐temporally modulated after injury or exercise to regulate muscle self‐renewal.

## 2 Results

### 2.1 Injury Transiently Induced Specific Prostanoids in Skeletal Muscles

**Figure 1:**
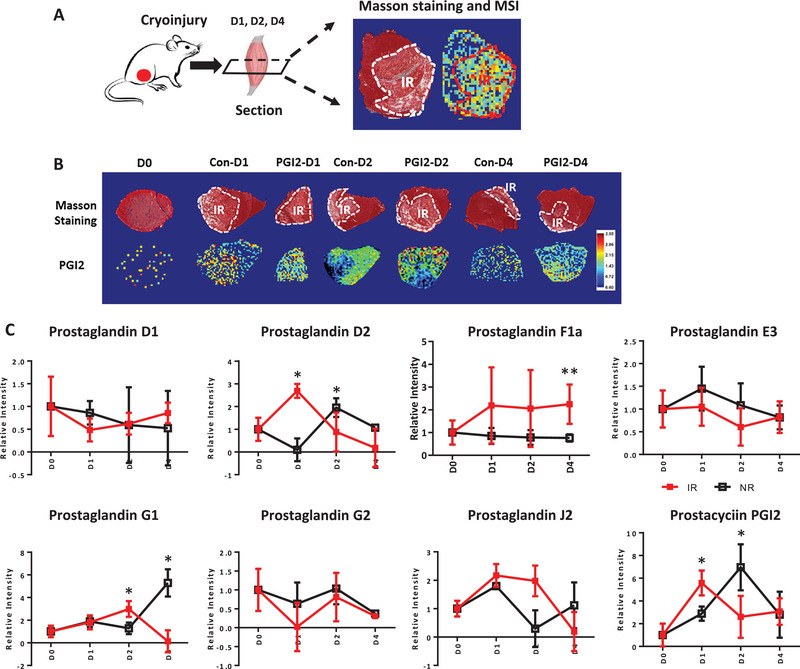
Some prostanoids increase transiently in regenerating muscles after injury. A) Schematic for cryoinjury of the TA muscle in young mice. Muscles were harvested on days 1–4 after injury for mass spectrometry imaging (MSI) and Masson trichrome staining. B) Masson trichrome staining and representative MALDI‐MSI images of the spatial distributions of PGI2 in TA muscles (*N* = 6 mice) at different days (1‐4) of regeneration after cryoinjury, without (Con) or with PGI2 intramuscular injection (*N* = 3 each). C) Eight prostanoids’ abundance in the injured region (IR) and normal region (NR) in TA muscles (*N* = 3 mice) at different time‐points of regeneration after cryoinjury. In total 12 tissue sections were analyzed by MALDI‐MSI (*N* = 3 each, 100 × 100 µm pixel). All tissue sections were collected and analyzed in the same batch. * *p* < 0.05, ** *p* < 0.01, Data were expressed as relative intensity ± SEM. The non‐parametric Mann–Whitney U test was used. Raw data are presented in Tables 1 and 2 (Supporting Information).

To survey the myobolites that are induced during the earliest stages of skeletal muscle injury in vivo, we cryoinjured the tibialis anterior (TA) muscles of young adult mice in vivo, then isolated skeletal muscle cryosections at different time‐points to analyze kinetic changes in metabolism during muscle regeneration (**Figure** **1**A). For this purpose, we utilized MALDI‐MSI to analyze TA muscle cryosection, then compiled the MSI data for a time‐series analysis. At this spatial resolution, we achieved a balance between discriminating single cells but collecting insufficient ions for analysis, versus discriminating only injured regenerative regions but collecting sufficient metabolite ions for analysis. By comparing our MSI data with Masson trichrome staining of corresponding tissue sections (Figure 1B), we could identify regions‐of‐interest (ROI) in the cryosections that corresponded to injured regenerating muscles, or adjacent normal muscles. Our MSI results revealed that several, but not all, prostanoids spreaded across and transiently spiked in the injured ROI over the first 4 days (Figure 1C; Tables 1 and 2, Supporting Information), while the adjacent normal muscles often showed a delayed spike in the same prostanoid. We interpreted prostanoids that showed a transient spike then declined in the injured region, and a delayed spike in the normal region, as injury‐induced myobolites. These included PGD2, PGG1, and the prostacyclin PGI2 (Figure 1C). The prostacyclin PGI2 also showed the largest injury‐induced fold change.

To confirm that their identification and quantification were accurate, we used titrations of several pure prostanoid standards on both ITO slides and muscle tissue sections to calibrate our measurements. The standard calibration curves further demonstrated that our MALDI‐MSI technique could accurately identify and quantify prostanoids in skeletal muscle sections (Figure S1, Supporting Information).

### 2.2 Prostacyclin PGI2 promotes young and aged muscle regeneration and declines with aging

**Figure 2:**
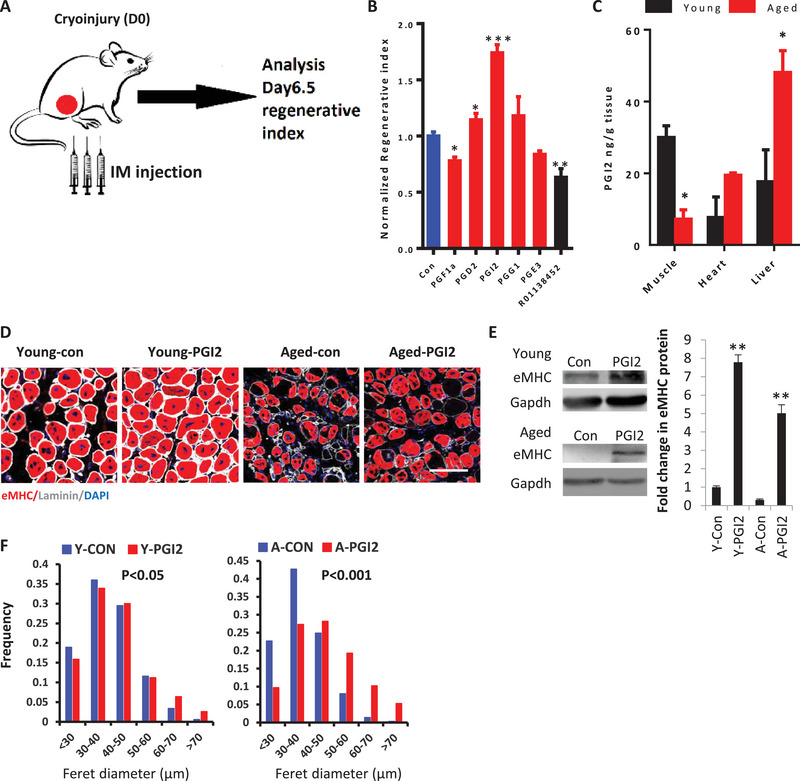
Prostacyclin PGI2 accelerates skeletal muscle regeneration and declines with aging in muscles. A) Schematic for cryoinjury of young mice’ TA muscle, followed by intramuscular injection of prostanoids or vehicle control (Con), and harvested at 6.5 days post‐injury for analysis of the regenerative index (% of myonuclei in eMHC+ myofibers). B) Quantification of the regenerative index (% of myonuclei in eMHC+ myofibers) of young TA muscles at 6.5 days after injury and treatment with PGF1a, PGD2, PGI2, PGG1, PGE3 or the PGI2 antagonist R01138452, relative to the vehicle control (Con). C) Prostacyclin PGI2 abundance in young and aged skeletal muscles, heart, and liver, as quantified by LC‐MS/MS (*N* = 3 mice each). D) Immunofluorescence staining for eMHC (red), 6 days after muscles were cryoinjured and injected with prostacyclin PGI2 or vehicle control (Con), in young mice and aged mice. Laminin(white); DAPI (blue). Scale bar, 50 µm. E) Western blot for eMHC protein expression in young and aged muscles in vivo (6 days post‐injury), after injection of prostacyclin PGI2, relative to the vehicle control (Con). Right panel: quantification of the western blots, normalized to Gapdh (*N* = 4 mice each). F) Distribution of myofiber diameters (Feret diameter) in the injured regions of vehicle control (Con)‐treated and PGI2‐treated muscles in young mice (left panel) and aged mice (right panel). *N* = 3 biological replicates unless mentioned otherwise. Data were expressed as mean ± SEM. Two‐tailed Student's t‐test was used in (B‐C) and (E‐F). *p < 0.05, ** *p* < 0.01, *** *p* < 0.001.

Even if myobolites showed spatio‐temporal kinetics that suggested injury‐induction, they might not have any functional effect on injury‐induced regeneration. Thus we tested several of these prostanoids by intramuscular injection into the injured muscles and analyzed them ≈6 days later (**Figure** **2**A). Immunofluorescence analysis of regenerating myofibers positive for embryonic myosin heavy chain (eMHC+) revealed that the prostacyclin PGI2 induced the most significant increase in muscle regenerative index (*p* < 0.001), followed by the prostaglandins PGD2 (*p* < 0.05) and PGG1 (*p* > 0.05). In contrast, the prostanoids which did not show injury‐induction kinetics, e.g. PGF1a and PGE3, all failed to increase the regenerative index (Figure 2B). Interestingly, the PGI2 antagonist R01138452 decreased the regenerative index significantly (*p* < 0.01), indicating that the prostacyclin PGI2 is both necessary and sufficient to promote skeletal muscle regeneration after injury. Furthermore we found that prostacyclin abundance decreases with aging, specifically in the skeletal muscles (Figure 2C), suggesting that prostacyclin's decline with aging might cause decreased muscle regeneration or self‐renewal, and thus sarcopenia.

Thus it was of interest to test if intramuscular supplementation of the prostacyclin PGI2 could improve both young and aged muscle regeneration. Immunofluorescence staining of skeletal muscle sections on day 6 showed that regenerating eMHC+ myofibers were larger and more numerous after PGI2 injection, in both young and aged 2‐year‐old mice (Figure 2D). Western blot analysis confirmed that intramuscular PGI2 injection increased eMHC protein expression levels in both young and aged muscles on day 6 (Figure 2E). Morphometric quantification further revealed that intramuscular PGI2 increased the myofiber diameter in young muscles (*p* < 0.05), and even more significantly for aged muscles (*p* < 0.001; Figure 2F). Altogether, these results indicated that intramuscular PGI2 supplementation is sufficient to accelerate both young and aged muscle regeneration.

### 2.3 Pax7+ MuSCs and MyoD+ myoblasts are activated by PGI2

**Figure 3:**
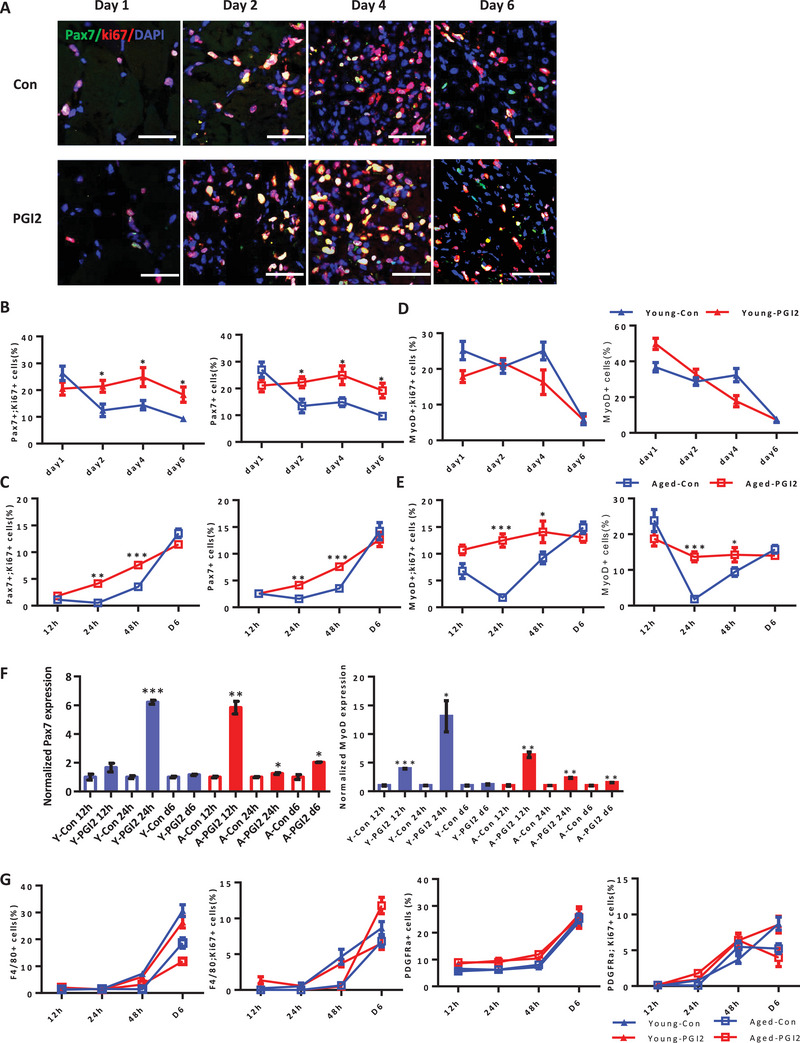
Pax7+ MuSCs and MyoD+ myoblasts are activated by the PGI2 spike in vivo. A) Representative images of Pax7+ (green) muscle stem cells and Ki67+(red) proliferative cells, at different time‐points of young muscle regeneration, after cryoinjury and intramuscular injection of PGI2 or DMSO vehicle control (Con); DAPI (blue). Scale bar, 50 µm. B) Quantification of the percentage of proliferative Pax7+Ki67+ muscle stem cells and Pax7+ muscle stem cells per field, at different time‐points of muscle regeneration, after cryoinjury and intramuscular injection of PGI2 or DMSO vehicle control in young mice (*N* = 3). C) Quantification of the percentage of proliferative Pax7+Ki67+ muscle stem cells and Pax7+ muscle stem cells per field, at different time‐points of muscle regeneration, after cryoinjury and intramuscular injection of PGI2 or DMSO vehicle control in aged mice (*N* = 3). D) Quantification of the percentage of proliferative MyoD+Ki67+ myoblasts and MyoD+ myoblasts per field, at different time‐points of muscle regeneration, after cryoinjury and intramuscular injection of PGI2 or DMSO vehicle control in young mice. E) Quantification of the percentage of proliferative MyoD+Ki67+ myoblasts and MyoD+ myoblasts per field, at different time‐points of muscle regeneration, after cryoinjury and intramuscular injection of PGI2 or DMSO vehicle control in aged mice. F) Quantification of Pax7 and MyoD protein expression in young and aged muscles (12, 24 h, or 6 days post‐injury) after injection of PGI2 post‐injury, relative to the DMSO vehicle control (Con). G) Quantification of the percentage of committed F4/80+ and proliferative F4/80+ Ki67+ macrophages, and PDGFRa+ fibroadipogenic progenitors (FAPs) per field, at different time‐points of muscle regeneration, after cryoinjury and intramuscular injection of PGI2 or DMSO vehicle control in young and aged mice. Data were expressed as mean ± SEM. Two‐tailed Student's *t*‐test was used in (B‐G). *p < 0.05, ** *p* < 0.01, *** *p* < 0.001, *N* = 3 biological replicates unless mentioned otherwise.

To identify the cells that are responsive to intramuscular PGI2's effects on skeletal muscle regeneration, we performed immunofluorescence staining for MuSCs, myoblasts, and non‐myogenic cells after intramuscular PGI2 injection. Our staining showed that Pax7+ MuSCs were significantly increased after PGI2 injection from day 2 onwards, including the proliferative and committed Pax7+ Ki67+ muscle stem cells in young mice (**Figure** **3**A,B). Similarly in aged muscles, both Pax7+ and Pax7+ Ki67+ MuSCs were significantly increased by 24 and 48 h after PGI2 injection, but the differences were no longer noticeable by day 6 compared to the control group (Figure 3C and Figure S2B, Supporting Information). Even though young MyoD+ myoblasts did not show significant changes after PGI2 injection (Figure 3D and Figure S2A, Supporting Information), aged MyoD+ myoblasts were significantly more resistant to cell death by 12 h after cryoinjury in mice injected with PGI2, relative to the control group (Figure 3E and Figure S2C, Supporting Information). Western blots of cryoinjured muscles further revealed that PGI2 promoted Pax7 protein expression at 24 h in young mice and from 12 h to day 6 in aged mice. PGI2 promoted MyoD protein expression from 12 to 24 h (Figure 3F and Figure S2D, Supporting Information) in both young and aged muscles. In contrast, both macrophages (F4/80+ cells) and fibro‐adipogenic precursors (PDGFRa+) did not show any significant changes throughout the 6 days of muscle regeneration after PGI2 injection (Figure 3G and Figure S3A,B, Supporting Information), indicating that Pax7+ MuSCs and MyoD+ myoblasts are the target cells most prominently activated by a PGI2 spike.

### 2.4 PGI2 Promotes Myoblast Proliferation, Early Differentiation, and PPAR*γ*/PGC1a Signaling

**Figure 4:**
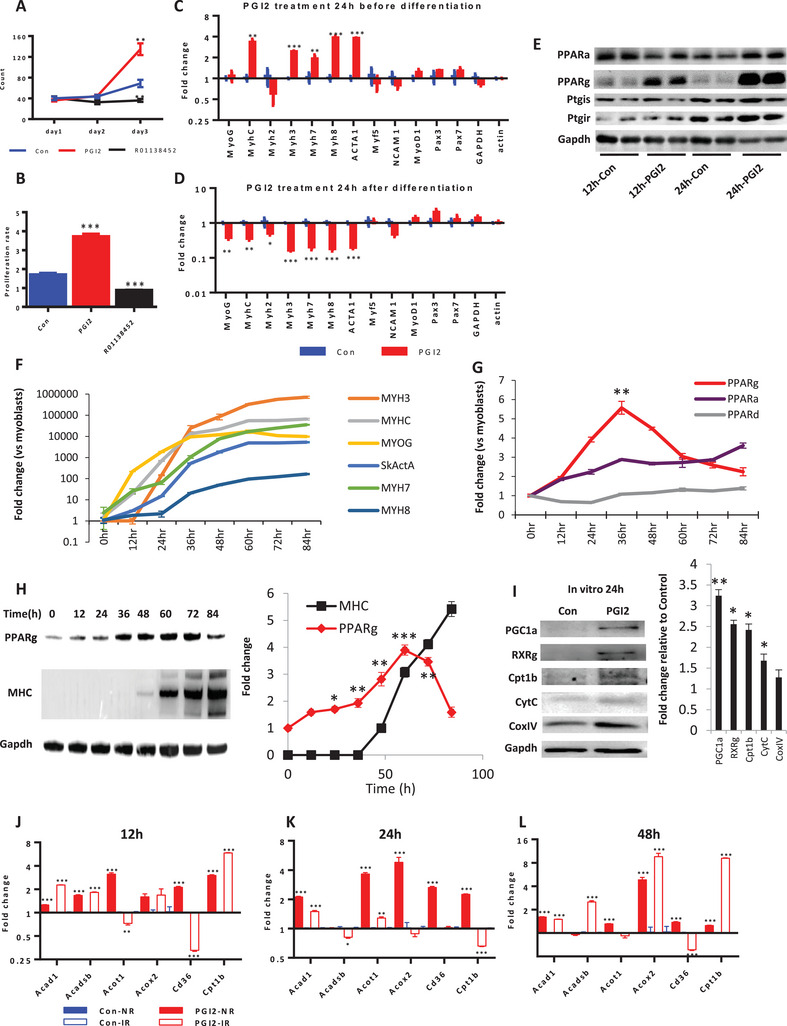
The PGI2 spike promotes MuSC proliferation, early differentiation, and a spike in PPARg/PGC1a signaling. A) Cell proliferation curves of 100% MyoD+ primary myoblasts after treatment with PGI2 and the PGI2 receptor antagonist R01138452, relative to the DMSO vehicle control (Con). B) Quantification of the proliferation rate by analyzing the proportion of Ki67+ myoblasts. C) Relative mRNA expression levels of myogenic and terminal differentiation markers when primary myoblasts were treated with PGI2 before differentiation. D) Relative mRNA expression levels of myogenic and terminal differentiation markers (normalized to Gapdh) when primary myoblasts were treated with PGI2 24 h after serum withdrawal and differentiation. E) Western blot for expression of PPARa, PPARg, PGI2 synthase (Ptgis) and PGI2 receptor (Ptgir) protein in TA muscle at 12 and 24 h post‐injury after intramuscular injection of PGI2, relative to the DMSO vehicle control (Con). F) Relative mRNA expression levels of myogenic differentiation markers (normalized to Gapdh) over the 84 h time‐course of primary myoblast differentiation. G) Relative mRNA expression levels of PPARa, PPARd and PPARg (normalized to Gapdh) over the 84 h time‐course of primary myoblast differentiation. H) Expression of PPARg and myosin heavy chain (MHC) protein over the 84 h time‐course of primary myoblast differentiation. Right panel: quantification of Western blot replicates, showing statistical significance of PPARg expression at different time‐points relative to 0 h. I) Western blot of lipid metabolism proteins (PGC1a, RXRg) and OxPhos‐related proteins (Cpt1b, cytochrome C, cytochrome oxidase IV) in primary myoblasts, after 24 h treatment with PGI2 or DMSO vehicle control (Con). Right panel: quantification of Western blot replicates, relative to vehicle control, normalized to Gapdh. J) Relative mRNA expression levels of FAO‐related genes (normalized to Gapdh) in the normal region (NR) and injured region (IR) of muscles at 12 h post‐injury after injection of PGI2 or DMSO vehicle control. K) Relative mRNA expression levels of FAO‐related genes (normalized to Gapdh) in the normal region (NR) and injured region (IR) of muscles at 24 h post‐injury after injection of PGI2 or DMSO vehicle control. L) Relative mRNA expression levels of FAO‐related genes (normalized to Gapdh) in the normal region (NR) and injured region (IR) of muscles at 48 h post‐injury after injection of PGI2 or DMSO vehicle control. Data were expressed as mean ± SEM. Two‐tailed Student's *t*‐test was used in (A–D) and (F–L). *p < 0.05, ** *p* < 0.01, *** *p* < 0.001, *N* = 3 biological replicates unless mentioned otherwise.

To accurately determine whether PGI2 has a direct effect on myoblast proliferation, we assessed the viability of pure MyoD+ myoblasts isolated by FACS^[^ ^9^ ^]^ (Figure S4A, Supporting Information), after treatment with PGI2 in vitro. We found that exposure to PGI2 in culture induced a threefold increase in the number of myoblasts, relative to the vehicle control, within 3 days (**Figure** **4**A). This increase in cell division after PGI2 treatment was also evident by Ki67 staining for mitotic cells (Figure 4B). In contrast, the PGI2 antagonist R01138452 significantly suppressed myoblast proliferation (Figure 4A,B). When treatment with PGI2 was performed before differentiation, several myogenic and terminal differentiation markers were induced (Figure 4C). However, when PGI2 treatment was performed 24 h after serum withdrawal and differentiation, many of these terminal differentiation markers were suppressed (Figure 4D). Taken together, these results suggest that a PGI2 spike promotes myoblast proliferation and commitment to the early phase of differentiation.

To explore the downstream mechanism for PGI2's effects on skeletal muscle regeneration, we examined the protein expression of several putative targets of PGI2 in the skeletal muscles during the 24 h post‐cryoinjury time window, after PGI2 injection. Western blot analysis revealed that PGI2 synthase (Ptgis) increased from 12 to 24 h post‐injury. Amongst the potential targets of PGI2, such as the IP receptor (Ptgir) and the PPAR nuclear receptors, intramuscular PGI2 induced PPAR*γ* protein expression most strongly, followed by PPAR*α* (Figure 4E).

To verify if the PPAR receptors are expressed during myogenesis in a manner that fits PGI2 spiking during early regeneration, our profiling of pure MyoD+ myoblasts (Figure S4A, Supporting Information) over a differentiation time‐course (Figure 4F), revealed that PPAR*γ* mRNA underwent a transient spike from 0 to 48 h of myogenesis during the earliest stage of differentiation, but declined back to basal levels by 84 h (Figure 4G). In contrast, PPAR*α* spiked and plateaued, then rose steadily for at least 84 h. PPAR*δ* mRNA did not change significantly during the differentiation of myoblasts (Figure 4G). Western blots confirmed that PPAR*γ* protein transiently spiked like PGI2 during the early phase of myogenic differentiation, and fell back to basal levels by 60–84 h (Figure 4H).

The PPAR*γ*/PGC1a (PPAR*γ* coactivator 1a) axis transactivates various genes to control lipid metabolism. To verify if PGI2's induction of PPAR*γ* could modulate lipid metabolism, we analyzed the expression of various PPAR targets in lipid metabolism. Our results showed that human myoblasts significantly upregulated PGC1a, RXRg, as well as carnitine palmitoyltransferase 1b (Cpt1b), the rate‐limiting enzyme for muscle fatty acid oxidation (FAO), and the mitochondrial OxPhos protein cytochrome C (Figure 4I). Moreover, mRNA expression revealed that PGI2 markedly increased the expression of many PPAR*γ*/PGC1a targets in FAO, including mitochondrial medium‐chain acyl‐CoA dehydrogenase (Acad1), mitochondrial short/branched chain acyl‐CoA dehydrogenase (Acadsb), acyl‐CoA thioesterase 1 (Acot1), acyl‐CoA oxidase 2 (Acox2), fatty acid translocase (CD36) and Cpt1b (Figure 4J–L), in cryoinjured muscles in vivo, especially in the injured region at 48 h (Figure 4L). Taken together, these data suggested that the PGI2 spike activated a PPAR*γ*‐PGC1a signaling network to stimulate mitochondrial FAO for myoblast proliferation and early differentiation.

### 2.5 PPAR*γ*/PGC1a‐FAO is Necessary and Sufficient to Control Myogenesis

**Figure 5:**
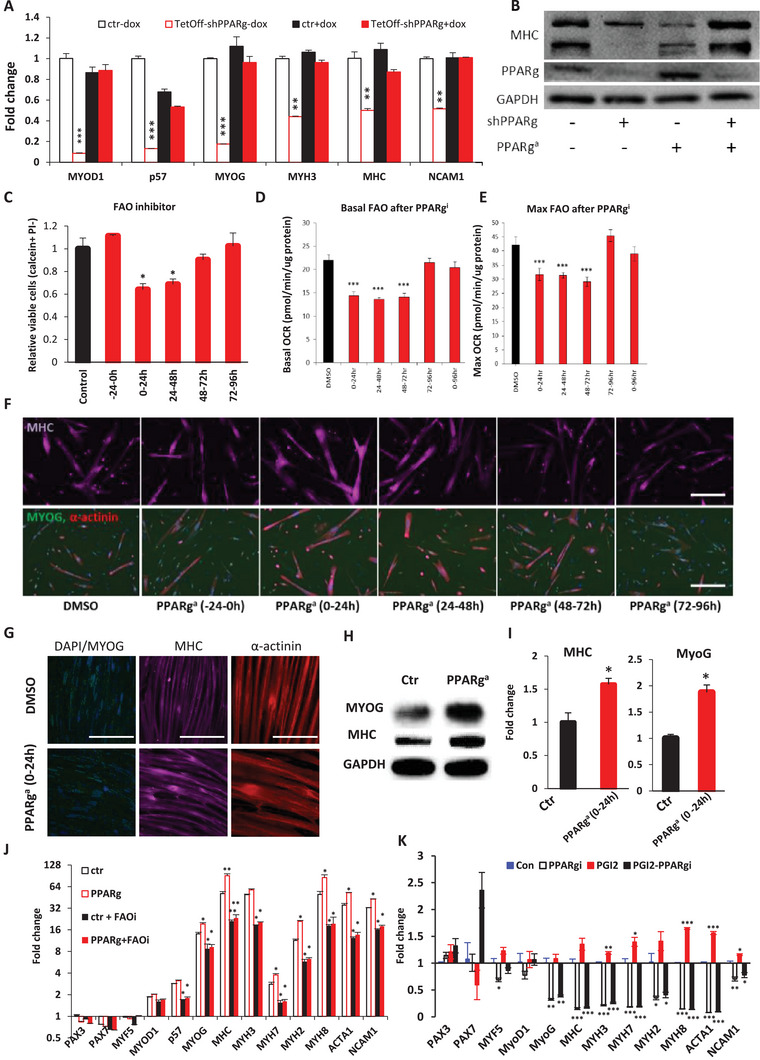
The PPARg/PGC1a‐FAO spike is required for early myogenic differentiation. A) Relative mRNA expression levels of myogenic markers (normalized to Gapdh) before and after TetOff doxycycline (dox)‐repressible shRNA for the transient knockdown of PPARg (shPPARg) during the first 48 h of differentiation, relative to empty vector control (ctr). B) Western blot of the differentiation marker myosin heavy chain (MHC) in myocytes, before and after TetOff doxycycline (dox)‐repressible shRNA for the transient knockdown of PPARg (shPPARg) during the first 48 h of differentiation, with or without PPARg agonist (PPARg^a^). C) Relative cell numbers after treating primary myocytes with the CPT1‐specific FAO inhibitor (FAOi) etomoxir (5 µm) during different time‐windows of myogenic differentiation. D) Basal oxygen consumption rate (OCR) in primary myocytes after inhibition with a PPARg inhibitor during different time‐windows in myogenic differentiation. E) Maximal OCR in primary myocytes after inhibition with a PPARg inhibitor during different time‐windows in myogenic differentiation. F) Immunofluorescence staining for the differentiation markers myosin heavy chain protein (purple), *α*‐actinin (red), and nuclear myogenin (green) proteins, after treatment with 10 µm PPARg agonist during different time‐windows in myogenic differentiation at low cell density conditions. Scale bar, 100 µm. G) Immunofluorescence staining for the myogenic differentiation markers: myosin heavy chain (MHC; purple), *α*‐actinin (red), and nuclear myogenin (green) proteins, after treatment with 10 µm PPARg agonist at the early 0–24 h time‐window in myogenic differentiation at high cell density conditions. Scale bar, 200 µm. H) Western blot of myogenin (MyoG) and myosin heavy chain (MHC) protein expression after treatment with 10 µm PPARg agonist at the early 0–24 h time‐window in myogenic differentiation at high cell density conditions. Scale bar, 200 µm. I) Quantification of the terminal differentiation markers MyoG and MHC after treatment with 10 µm PPARg agonist at the early 0–24 h time‐window in myogenic differentiation at high cell density, relative to the vehicle control (Ctr). J) mRNA expression profile of myogenic factors and markers in myocytes, after lentiviral overexpression of PPARg, relative to the empty vector control (ctr), with and without the FAO inhibitor (FAOi) etomoxir (5 µm) for 48 h. K) mRNA expression profile of myogenic factors and markers in myocytes, after treatment with PGI2, relative to the empty vector control (Con), with and without the PPARg inhibitor (PPARgi) (10 µm) for 48 h. Data were expressed as mean ± SEM. Two‐tailed Student‘s *t*‐test was used in (A), (C–E), and (I–K). *p < 0.05, ** *p* < 0.01, *** *p* < 0.001. *N* = 3 biological replicates unless mentioned otherwise.

To further test if PPAR*γ*/PGC1a is necessary for normal myogenesis, we used a TetOff shRNA to knockdown PPAR*γ* expression in pure human myoblasts only at required times (**Figure** **5**A). Our results showed that the expression of many myogenic factors and markers, including MyoD (MYOD1), p57, myogenin (MYOG), embryonic myosin heavy chain (eMHC, MYH3), pan‐myosin heavy chain (MHC), and NCAM1, were significantly suppressed upon PPAR*γ* knockdown during early differentiation (Figure 5A), indicating that PPAR*γ* is required for normal myogenesis. This was further supported by Western blots showing that PPAR*γ* knockdown suppressed myosin heavy chain (MHC) protein expression, and this was reversible by the PPAR*γ*‐specific agonist rosiglitazone at target‐specific, low µm concentrations (Figure 5B).

We also applied the CPT1‐specific inhibitor etomoxir at target‐specific, low µm concentrations^[^ ^10^ ^]^ to test if mitochondrial FAO was indeed necessary during different time‐windows of myoblast differentiation. Interestingly, we found that mitochondrial FAO inhibition compromised myocyte viability at 0–24 h and 24–48 h of myoblast differentiation (Figure 5C), but not later time‐windows, suggesting a transient but specific requirement for mitochondrial FAO only at 0–48 h of myogenic differentiation.

To test if FAO itself is also necessary for normal myogenic differentiation, the surviving myocytes remaining after FAO inhibition were assayed for changes in myogenic markers by Western blot. We found that mitochondrial FAO inhibition at different time‐windows led to different profiles of myogenic markers (Figure S4B, Supporting Information). Semi‐quantitative analysis of the Western blots led us to conclude that 0–24 h mitochondrial FAO inhibition caused an MHC^low^; MYOG^low^ phenotype, indicating that myogenic differentiation was blocked as a whole. Twenty‐four to 48 h mitochondrial FAO inhibition caused an MHC^low^; MYOG^high^ phenotype, indicating that MYOG+ myocytes were now inhibited from fusing and differentiating into MHC+ myotubes. Fourty‐eight to 72 h mitochondrial FAO inhibition caused an MHC^high^; MYOG^low^ phenotype (Figure S4C, Supporting Information), indicating that MYOG+ myocytes could fuse and differentiate into MHC+ myotubes more efficiently, but MYOG+ myocytes were being depleted prematurely. Seventy‐two to 96 h mitochondrial FAO inhibition caused an MHC^high^; MYOG^high^ phenotype (Figure S4C, Supporting Information), indicating that late‐stage mitochondrial FAO inhibition actually enhanced myogenic differentiation instead. Taken together, our results indicate that the spike in PGI2‐PPAR*γ*/PGC1a‐FAO is necessary to promote early myoblast differentiation, but suppresses terminal differentiation.

By carefully applying a PPAR*γ* inhibitor over a series of time‐windows in the presence of palmitate, we tested if PPAR*γ* was the driving mechanism for regulating the early transient burst of FAO‐fueled OxPhos. Our results revealed that PPAR*γ* inhibition at 0–24, 24–48, and 48–72 h could significantly reduce both basal and maximal oxygen consumption rates in fatty acid‐fed myocytes, but not thereafter from 72 to 96 h (Figure 5D,E). Thus, PPAR*γ* is necessary for mitochondrial FAO during early myogenic differentiation. Interestingly, the PPAR*γ* inhibitor failed to reduce the oxygen consumption rate when applied throughout myogenesis (0–96 h), suggesting that compensatory responses are triggered when a PPAR is chronically inhibited for too long (Figure 5D,E).

Having established that the transient spike in PPAR*γ*/PGC1a‐FAO was necessary for myoblast differentiation, it was of interest to test if PPAR*γ*/PGC1a‐FAO was also sufficient for promoting myoblast differentiation. We tested the effects of the PPAR*γ*‐specific agonist rosiglitazone on myocytes, at 48 h after induction of human myoblast differentiation. RNAseq confirmed that a total of 321 genes were significantly upregulated and 279 genes were significantly downregulated (Figure S4D, Supporting Information). Gene Set Enrichment Analysis (GSEA) confirmed that PPAR*γ* activation promoted the “Fatty Acid Beta Oxidation” (FAO) signature at the genome‐wide level (Figure S4E, Supporting Information, *p* < 0.01), and that PPAR*γ*‐FAO activation was sufficient to promote the “Eukaryotic Translation Elongation” ribosomal signature associated with myogenic growth (Figure S4F, Supporting Information, *p* < 0.001), and the “Striated Muscle Contraction” myogenesis signature (Figure S4G, Supporting Information, *p* = 0.031).

Further testing showed that PPAR*γ* activation at the 0–24 h time‐window uniquely upregulated the mRNA levels of myogenin (MYOG), adult type I myosin heavy chain (MYH7), and perinatal myosin heavy chain (MYH8) in pure myocytes, whereas other time‐windows of treatment had no significant effects (Figure S5A–C, Supporting Information). When the PPAR*γ*‐activated myocytes were immunostained for the myogenesis markers MHC protein and *α*‐actinin, it was clear that PPAR*γ* activation at the 0–24 and 24–48 h time‐windows significantly enhanced myogenesis at low density (Figure 5F). When we repeated PPAR*γ* activation on human myocytes at high density in this optimal 0–24 h window, we found that the resultant human myotubes showed significantly more hypertrophic growth than the control human myotubes (Figure 5G). Quantification of MYOG and MHC protein expression by Western blots further confirmed these observations (Figure 5H,I).

When PPAR*γ* was overexpressed, several myogenic factors and markers were induced at the 48 h time‐point of early myoblast differentiation (Figure 5J). However, FAO inhibition significantly suppressed many of these changes, confirming that PPAR*γ* is sufficient to induce early myogenic differentiation in an FAO‐dependent manner (Figure 5J). Furthermore, PGI2's effect on early myogenic differentiation was reversed by a PPAR*γ* inhibitor (Figure 5K). Thus, the PGI2‐PPAR*γ*/PGC1a‐FAO axis is necessary and sufficient to promote early myogenic differentiation.

### 2.6 Mitochondrial FAO During Early Myoblast Differentiation In Vitro and In Vivo

**Figure 6:**
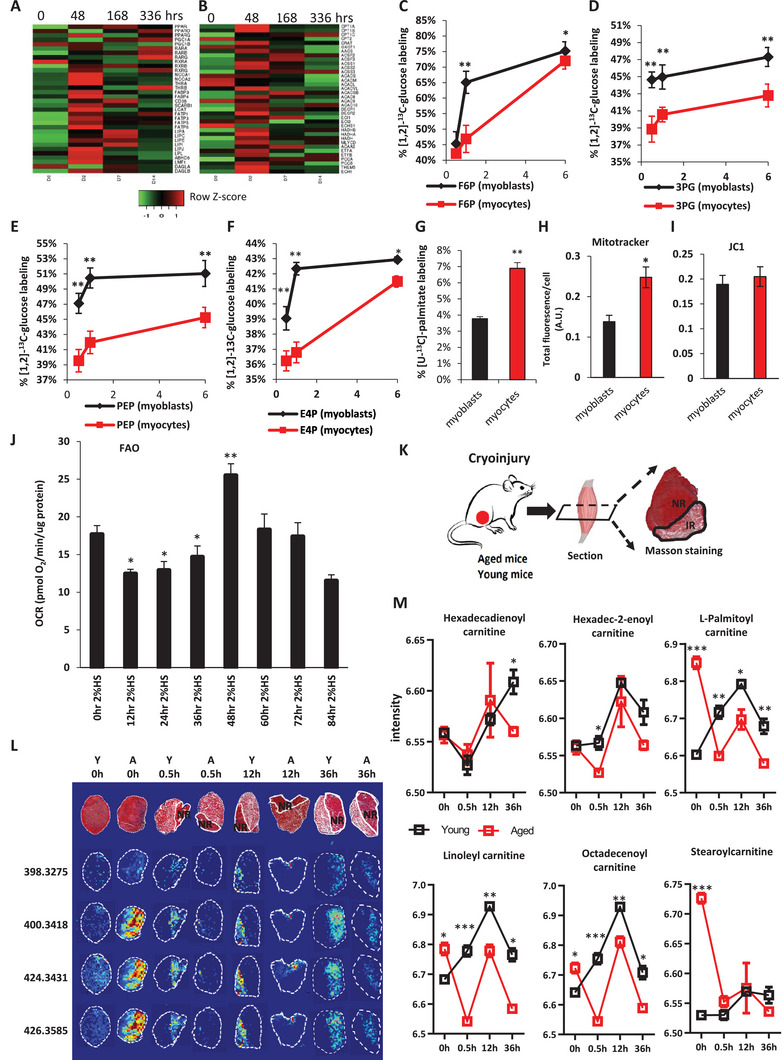
FAO spikes during early muscle regeneration, but becomes delayed upon aging in vivo. A) Relative mRNA expression levels of upstream regulators of fatty acid metabolism, over a 14 d time‐course in human myoblast differentiation, according to GSE55034. B) Relative mRNA expression levels of downstream effectors of fatty acid metabolism, over a 14 d time‐course in human myoblast differentiation, according to GSE55034. C) [1, 2–^13^C] glucose flux into the glycolytic intermediate glucose/fructose‐6‐phosphate (F6P) in post‐mitotic mononucleated primary human myocytes after differentiation for 48 h. D) [1, 2–^13^C] glucose flux into the glycolytic intermediate 3‐phosphoglycerate (3PG) in post‐mitotic mononucleated primary human myocytes after differentiation for 48 h. E) [1, 2–^13^C] glucose flux into the glycolytic intermediate phosphoenolpyruvate (PEP) in post‐mitotic mononucleated primary human myocytes after differentiation for 48 h. F) [1, 2–^13^C] glucose flux into the glycolysis‐pentose phosphate pathway intermediate erythrose‐4‐phosphate (E4P) in post‐mitotic mononucleated primary human myocytes after differentiation for 48 h. G) [U‐^13^C] palmitate flux (6 h) into acetyl‐CoA in post‐mitotic mononucleated primary human myocytes after differentiation for 48 h. H) Quantification of mitochondrial volume and mitochondrial membrane potential in post‐mitotic mononucleated primary human myocytes after differentiation for 48 h, relative to primary human myoblasts, by fluorescence staining with Mitotracker Red. I) Quantification of mitochondrial volume and mitochondrial membrane potential in post‐mitotic mononucleated primary human myocytes after differentiation for 48 h, relative to primary human myoblasts, by fluorescence staining with JC1. J) Quantification of basal respiration rates in myocytes over the course of myogenic differentiation, by measuring basal oxygen consumption rates (OCR) in fatty acid‐supplemented differentiation media every 12 h for 84 h. K) Schematic for cryinjury of the TA muscle in young mice and >2‐year‐old geriatric mice, harvested at 0, 0.5, 12, or 24 h after injury for mass spectrometry imaging and Masson trichrome staining. L) Mass trichrome staining and representative MALDI‐MSI images of the spatial distributions of lipid metabolites in young and >2‐year‐old geriatric mice’ TA muscles, at different time‐points of regeneration after cryoinjury. Masson trichrome staining of the tibialis anterior (TA) muscle at different time‐points of muscle regeneration. M) Time‐course graphs of the abundance of each selected metabolite in the normal region (NR) of young and geriatric muscles (*N* = 3 each), after cryoinjury. In total 24 tissue sections were analyzed by MALDI‐MSI. All tissue sections were collected and analyzed in the same batch (100 × 100 µm pixels, *N* = 3). Log‐transformed average intensity values for each sample are shown. Data were expressed as mean ± SEM. Student's *t*‐test was used if all samples passed the Shapiro–Wilk normality test (*p* > 0.05) and Levene's homogeneity of variance test (*p* > 0.05); otherwise, the non‐parametric Mann–Whitney U test was used. *p < 0.05; **p < 0.01; ***p < 0.001. Statistical results are summarized in Tables 2 and 3 (Supporting Information). Data were expressed as mean ± SEM. Two‐tailed Student‘s t‐test was used in C‐J. * *p* < 0.05, ** *p* < 0.01, *** *p* < 0.001, *N* = 3 biological replicates unless mentioned otherwise.

To broadly survey the lipid metabolism changes during myogenesis, we mined transcriptomic data on primary human myoblast differentiation in the GEO database (GSE55034). We found that a variety of lipid metabolism and FAO‐related genes were indeed upregulated transiently after the initiation of primary human myoblast differentiation. These include the upstream transcriptional master regulators of lipid metabolism: the nuclear hormone receptors (PPARg, RXRg, RXRb, NCOA1, NCOA2), the upstream fatty acid transporters (FABP3, FABP4, CD36, SCARB1, FATP1‐6), and a variety of lipases including LPL (**Figure** **6**A). Furthermore, we also observed a transient upregulation of mitochondrial FAO enzymes by 48 h, including the rate‐limiting carnitine palmitoyl‐transferases (CPT1A and CPT1B), the carnitine acetylase CRAT, many acyl‐CoA dehydrogenases (ACADs), many hydroxyacyl‐CoA dehydrogenases (HADHs), and the mitochondrial electron transfer flavoproteins ETFA and ETFB, most of which are critical for mitochondrial FAO (Figure 6B). It should be noted that these trends largely disappeared by days 7–14 of primary human myoblast differentiation, confirming again that mitochondrial FAO is transcriptionally upregulated only during the early phase of primary myoblast differentiation.

To verify our findings on FAO during myogenic differentiation, we performed LC–MS/MS metabolomics profiling of pure MyoD+ human myoblasts (Figure S4A, Supporting Information), freshly isolated from normal human muscle biopsies by FACS.^[^ ^9^ ^]^ We found that 48 h of myogenic differentiation induced critical metabolic changes as the primary human myoblasts underwent a transition into non‐proliferative myocytes (Figure S6A, Supporting Information). As expected of 48 h myocytes in the early phase of myogenic differentiation, whereupon they activate cyclic AMP‐PKA signaling and the muscle‐specific creatine kinase^[^ ^11^ ^]^, we observed significant increases in cAMP, creatine and phosphocreatine (Figure S6B, Supporting Information). Coinciding with these myocyte‐specific metabolic changes, we also observed significant increases in several acylcarnitines, suggesting an upregulation of FAO (Figure S6C, Supporting Information). It should also be noted that despite the serum withdrawal during differentiation, which decreased albumin‐bound lipid and fatty acid‐fueled FAO, we still observed an increase in FAO. Thus these metabolic observations cannot be explained by changes in nutrient availability during serum withdrawal. Furthermore, LC–MS/MS revealed that redox‐associated metabolites increased as well, including oxidized glutathione, glutathione, and NADH (Figure S6D, Supporting Information). Comparing the oxidized glutathione/reduced glutathione ratio, and the NADH/NAD^+^ ratio, our results suggested an increase in both reducing equivalents and oxidative stress at the myocyte stage. This corroborates an increase in mitochondrial FAO during the early phase of myogenesis, since mitochondrial FAO increases both NADH/NAD^+^ and reactive oxygen species (ROS) very efficiently.^[^ ^12^ ^]^

While no significant changes were observed for several glycolytic intermediates at steady state (Figure S6E, Supporting Information), we postulated that an upregulation of FAO should lead to a drop in ^13^C‐glycolysis flux and an increase in ^13^C‐palmitate flux. To test this, we incubated equal numbers of myoblasts and myocytes in [1,2‐^13^C]‐glucose medium for 6 h (Figure 6C–F), to test for kinetic changes in flux through the glycolysis and pentose phosphate pathways.^[^ ^13^ ^]^ Our targeted LC‐MS/MS results showed that myogenic differentiation causes a significant decrease in ^13^C‐labeling of glucose/fructose‐6‐phospate (F6P). Using [1,2‐^13^C]‐glucose instead of [U‐^13^C]‐glucose allows us to distinguish between flux from glycolysis or the pentose phosphate pathway,^[^ ^13^ ^]^ to 3‐phosphoglycerate (3PG) and phosphoenolpyruvate (PEP), even though only half the pool is labeled at maximum. Our results indicated a decrease in glycolytic flux (Figure 6C–F). The ^13^C‐labeling kinetics of erythrose‐4‐phosphate (E4P) indicated that myoblasts’ high glucose flux into the pentose phosphate pathway was also downregulated after differentiation into myocytes (Figure 6F). In contrast, when we incubated equal numbers of myoblasts and myocytes in [U‐^13^C]‐palmitate medium for 6 h, ^13^C‐labeling of acetyl‐CoA was significantly increased after early myogenic differentiation into myocytes (Figure 6G). Thus, we confirmed that FAO flux is upregulated and glycolytic flux is downregulated in the early phase of myogenic differentiation.

FAO can occur in the mitochondria or non‐mitochondrial compartments. To test whether mitochondrial capacity is functionally increased during early myoblast differentiation, we stained the proliferative primary human myoblasts and 48 h non‐proliferative myocytes with Mitotracker Red and JC1 dyes, to examine their mitochondrial volume and membrane potential, respectively. We found that by 48 h of early differentiation, human myocytes manifested a significant increase in mitochondrial volume (Figure 6H and Figure S6F, Supporting Information). In contrast, we did not observe a significant change in mitochondrial membrane potential Δ*ψ*_m_ (Figure 6I). We checked the mitochondrial DNA copy number, but found no significant changes at this early phase of myogenesis (Figure S6G, Supporting Information), suggesting the early increase in mitochondrial volume was not due to an increase in mitochondrial replication, but due to an increase in mitochondrial mass.

Next, we turned to the Seahorse Analyzer to measure oxygen consumption rates in palmitate‐fed myocytes, to confirm if all these mitochondrial and metabolic changes translated to an increase in mitochondrial FAO activity. Our analyses revealed that both the basal and maximal oxygen consumption rate rose transiently over time as differentiation progressed (Figure 6J). The oxygen consumption rate peaked at ≈48 h after induction of differentiation, when myoblasts had committed into non‐proliferative myocytes. However, the palmitate‐fueled oxygen consumption rate dropped significantly after this transient spike, and continued in decline by 84 h of differentiation (Figure 6J). Thus, in support of our transcriptomic, metabolomic and microscopic findings, our Seahorse Analyzer results confirmed that mitochondrial FAO also spiked during early differentiation of pure myoblasts in vitro.

In order to survey the fatty acid‐related metabolites induced during early muscle regeneration in vivo, we again compared our MSI data with Masson trichrome staining (Figure 6K,L), especially at the regions‐of‐interest (ROI) that corresponded to regenerating muscle at the border of the necrotic region. Our results revealed that numerous acylcarnitine species were transiently increased in the regenerative muscle ROI from 12 to 36 h (Figure 6M). Given that acylcarnitines are indicators of the mitochondrial FAO capacity of muscle subtypes our results confirmed that skeletal muscles exhibited a spike in FAO flux during early regeneration in vivo.

In parallel, we also sampled >2‐year old geriatric aged mice, to metabolically analyze aged muscle regeneration at every time‐point by MSI (*N* = 3 each, Table 1, Supporting Information). Previous studies had shown that muscle regeneration declines while muscle fibrosis increases with aging.^[^ ^14^ ^]^ MSI analysis of aged TA muscles after cryoinjury showed that the transient spike of acylcarnitines we observed in young adult muscle regeneration, was significantly delayed in aged mice (Figure 6M, Tables 1‐2, Supporting Information). These results suggest that a delayed FAO spike is associated with the dysfunctional regeneration during aging. Interestingly several long‐chain acylcarnitines, such as palmitoyl‐, linoelaidyl‐, octadecenoyl‐ and stearoyl‐carnitine, were significantly higher in aged myofibers than young adult myofibers at the beginning (0 h, Table 3, Supporting Information), suggesting that there is already excessive inflammation‐induced FAO in aged myofibers even before injury (Figure 6M), similar to the situation in cachexia.^[^ ^15^ ^]^ Thus the spatio‐temporal kinetics of regeneration‐associated FAO is dysregulated during aging.

### 2.7 Enhancing Skeletal Muscle Regeneration via PPAR*γ*/PGC1a‐FAO In Vivo

**Figure 7:**
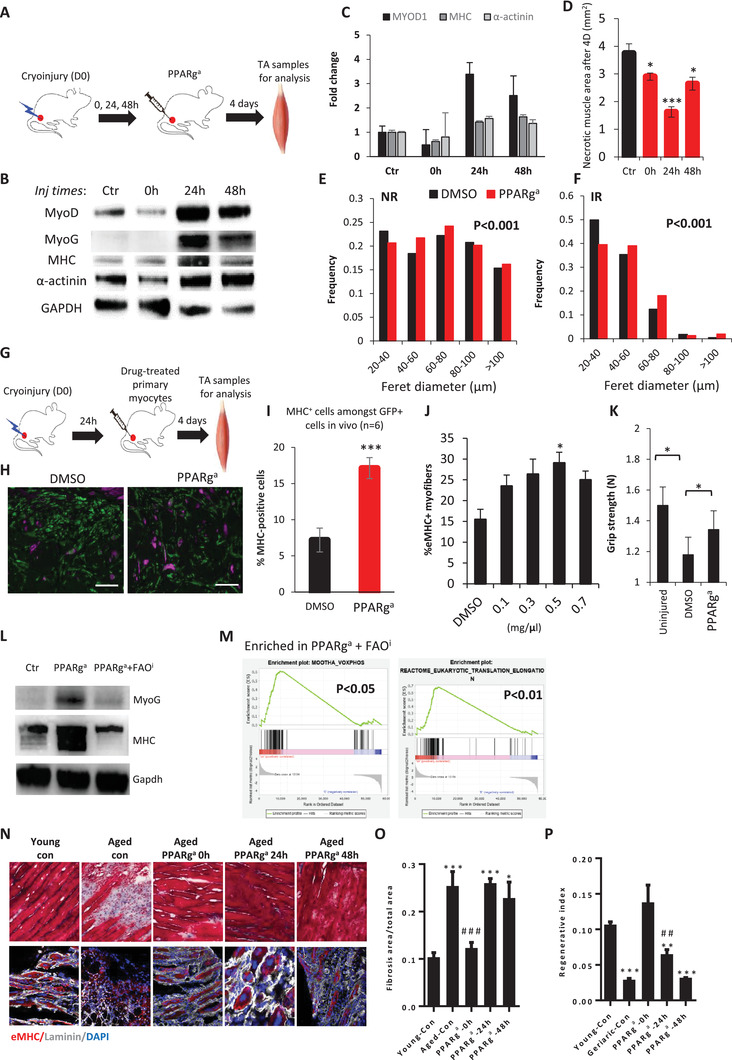
The PPARg‐FAO spike is necessary and sufficient to boost muscle regeneration in vivo. A) Schematic for cryoinjury of the TA muscle in young adult mice, followed by intramuscular injection of a single bolus of the PPARg agonist (PPARg^a^) at 0, 24, or 48 h after injury. TA muscles were harvested for analysis 4 days after injury. B) Western blot for the mouse differentiation markers MyoD, MyoG, MHC, and *α*‐actinin in the TA muscle (4 days post‐injury) after injection of a single bolus of the PPARg agonist (PPARg^a^) at 0, 24, or 48 h after injury, relative to a PBS vehicle control (Ctr). C) Quantification of the differentiation markers MyoD, MHC, and *α*‐actinin in the TA muscle (4 days post‐injury) after injection of a single bolus of the PPARg agonist (PPARg^a^) at 0, 24, or 48 h after injury, relative to a PBS vehicle control (Ctr). D) Quantification of the remaining necrotic muscle area in the TA muscle (4 days post‐injury) after injection of a single bolus of the PPARg agonist (PPARg^a^) at 0, 24, or 48 h after injury. E) Feret diameter distributions of myofibers in PBS vehicle control and PPARg^a^‐treated TA muscles in the normal region (NR). *N* = 4. F) Feret diameter distributions of myofibers in PBS vehicle control and PPARg^a^‐treated TA muscles in the injured region (IR). *N* = 4. G) Schematic for cryoinjury of the TA muscle in mice, followed by intramuscular injection of a single bolus of GFP+ human myocytes treated with PPARg^a^ or DMSO vehicle control, 24 h after injury. TA muscles were harvested for analysis 4 days after injury. H) Representative images of MHC+ cells (purple) amongst the GFP+ human myocytes, treated with PPARg^a^ or the DMSO vehicle control, that engrafted into the cryoinjured TA muscle 4 days post‐injury. I) Quantification of differentiated MHC+ cells amongst the GFP+ human myocytes that engrafted into the cryoinjured TA muscle 4 days post‐injury. J) Quantification of embryonic MHC‐positive (eMHC+) myofibers after titrating increasing doses of PPARg^a^. K) Assessment of grip strength after cryoinjury and treatment with PPARg^a^, relative to the DMSO vehicle control. L) Western blot of the myogenic markers MyoG protein and MHC protein, after cryoinjury and treatment with PPARg^a^ or PPARg^a^ plus an FAO inhibitor (PPARg^a^+FAOi), relative to vehicle control (Ctr). TA muscles were harvested for analysis 4 days after injury. M) Mitochondrial oxidative phosphorylation (OxPhos) signature was significantly downregulated after treatment with PPARg^a^+FAOi, relative to PPARg^a^ alone (left panel). The eukaryotic ribosomal translation elongation signature, associated with muscle growth, was significantly downregulated after treatment with PPARga+FAOi, relative to PPARg^a^ alone (right panel). N) Cryoinjury of the TA muscle in >2 year‐old aged mice, followed by intramuscular injection of a single bolus of PPARg^a^ at 0, 24, or 48 h after injury. TA muscles were needle biopsied for analysis of the regenerative index 6 days after injury, and harvested for analysis of fibrosis 27 days after injury. Top row: representative images of Masson trichrome staining of TA muscle sections at 27 days after injury; Bottom row: representative images of embryonic myosin heavy chain (eMHC(red)) and laminin(white) co‐immunofluorescence staining of TA muscle biopsies at 6 days after injury. O) Quantification of the ratio of fibrotic area to the total area of TA muscle sections in (N). ### *p* < 0.001, relative to Aged control, * *p* < 0.05, ** *p* < 0.01, *** *p* < 0.001, relative to Young control. P) Quantification of the regenerative index of TA muscle biopsies at 6 days after injury, from young and aged mice treated with DMSO vehicle control, or aged mice treated with PPARg^a^ at 0, 24, or 48 h after injury. Data were expressed as mean ± SEM. Two‐tailed Student‘s t‐test was used in (A), (C–F), and (I,J), One‐way ANOVA with Bonferroni's post hoc test was used in (K) and (O‐P). * *p* < 0.05, ** *p* < 0.01, *** *p* < 0.001, *N* = 3 biological replicates unless mentioned otherwise.

After finding that a PPAR*γ*/PGC1a‐FAO spike is necessary for early myoblast differentiation in vitro, and that regeneration‐associated FAO is dysregulated during muscle aging in vivo, we asked if PPAR*γ*/PGC1a‐FAO could be appropriately upregulated to enhance muscle regeneration in vivo. We injected a single bolus of PPAR*γ*‐specific agonist (with a short half‐life of ≈3 h) into the TA muscles at 0, 24, or 48 h after cryoinjury. 4 days after the injection phase, the muscle was harvested. Western blot analysis showed that intramuscular PPAR*γ* activation at 24 h post‐cryoinjury elicited the strongest expression of MyoD and MyoG protein (**Figure** **7**B,C). PPAR*γ* activation at both 24 and 48 h resulted in stronger expression of several MHC protein isoforms and *α*‐actinin protein levels, relative to the DMSO vehicle control and 0 h time‐window (Figure 7B,C). Quantification of the necrotic muscle area confirmed that 24 and 48 h injection of the PPAR*γ* agonist improved skeletal muscle regeneration in vivo (Figure 7D). Morphometric quantification further revealed that PPAR*γ* agonist increased the myofiber diameter in both the non‐injured region (NR) and injured region (IR) (Figure 7E,F).

To assess if these findings are relevant to human myoblasts^[^ ^16^ ^]^ and to test if the effects of PPAR*γ* activation are specific to myoblasts instead of other cell‐types, we pre‐treated pure GFP+ human myoblasts that were initiated into myogenic differentiation with either DMSO vehicle or the PPAR*γ* agonist for the 0–24 h time‐window, then orthotopically injected the GFP+ human myoblasts into the TA muscle of immunodeficient mice 24 h after cryoinjury (Figure 7G). Our immunofluorescence analysis results showed that transient activation of PPAR*γ* in myoblasts significantly enhanced MHC expression among the engrafting GFP+ human myocytes (Figure 7H,I), indicating that transient PPAR*γ* activation can enhance myogenesis after MTT. Furthermore, the fraction of eMHC+ myofibers was significantly increased by the PPAR*γ* agonist in a dose‐dependent manner (Figure 7J), suggesting that the PPAR*γ* agonist can directly enhance myoblast‐mediated regeneration in vivo. Indeed, 7 days after injury, the grip strength of the PPAR*γ* agonist‐treated mice was significantly higher than the vehicle control‐treated mice (Figure 7K).

To confirm that these effects in vivo were dependent on the PPAR*γ*/PGC1a‐FAO axis, we tested if the FAO inhibitor etomoxir could abrogate the PPAR*γ* agonist's positive effects. Our results revealed that the pro‐myogenic MyoG and MHC protein expression induced by PPAR*γ* was abrogated by mitochondrial FAO inhibition (Figure 7L). From the genome‐wide RNAseq perspective, etomoxir also suppressed the mitochondrial OxPhos signature and the “Eukaryotic Translation Elongation” ribosomal signature associated with myogenic growth (Figure 7M). Altogether, these results indicated that a transient spike in PPAR*γ*/PGC1a‐FAO induces early myoblast differentiation and thus early muscle regeneration in vivo.

Next, we sought to test if PPAR*γ*/PGC1a‐FAO upregulation could improve aged muscle regeneration. After cryoinjury of the TA muscles of >2‐year‐old geriatric mice, we intramuscularly injected a single bolus of the PPAR*γ* agonist at different time‐points, then assessed the regeneration and fibrosis of the TA muscles relative to young adult mice. As expected, Masson trichrome staining showed that the old mice had increased fibrosis after muscle regeneration, compared to young adult mice (Figure 7N,O). Intramuscular PPAR*γ* activation at the early 0 h time‐point significantly reduced fibrosis, compared to the old DMSO control, the 24 h and the 48 h time‐points (Figure 7O). Consistently, we found that at the early 0 h time‐point, a single PPAR*γ* agonist injection significantly improved the old muscles’ fraction of eMHC+ myofibers (Figure 7P). In contrast, intramuscular PPAR*γ* activation at the 24 and 48 h time‐points did not show as strong an improvement in the fraction of eMHC+ myofibers. Moreover, at 7 days after injury, the grip strength of mice treated with the PPAR*γ* agonist was significantly higher than mice treated with the vehicle control (Figure S7A, Supporting Information). The single bolus of PPAR*γ* agonist also had no effect on total body weight (Figure S7B, Supporting Information), although it significantly increased acylcarnitines (Figure S7C, Supporting Information) and thus FAO flux (Figure S7C, Supporting Information). Thus, these results confirmed that a PPAR*γ*/PGC1a‐FAO spike can promote aged muscle regeneration, but only specifically when administered at the earliest phase.

### 2.8 PGI2 Requires PPAR*γ* Activity to Promote Aged Muscle Regeneration and Function

**Figure 8:**
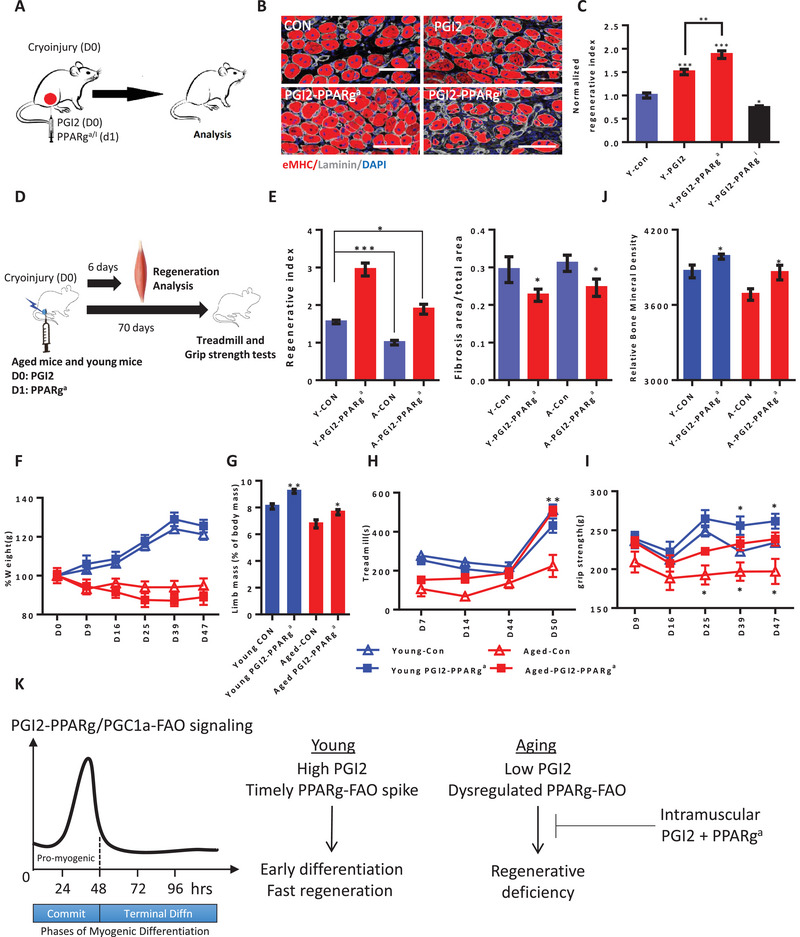
Spikes of prostacyclin and PPARg/PGC1a‐FAO agonist synergize to restore aged muscle regeneration in vivo. A) Schematic for cryoinjury of the TA muscle in young adult mice, followed by intramuscular injection of PGI2 and the PPARg agonist (PPARg^a^) or PPARg inhibitor T0070907 (PPARg^i^) at 0 and 24 h after injury, respectively. Muscles were harvested for analysis 6 days after injury. Data were from at least three independent experiments. B) Immunofluorescence staining for eMHC, a molecular marker of newly forming myofibers in regenerating muscles, 6 days after cryoinjury of muscles. Scale bar, 50 µm. Data were from at least three independent experiments. C) Quantification of the regenerative index of muscles at 6 days after injury, from young mice treated with PGI2 and PPARg^a^ or PPARg^i^ after injury, relative to the DMSO vehicle control. Data were from at least three independent experiments. D) Schematic for cryoinjury of muscles in >18 month‐old aged mice, followed by intramuscular injection of PGI2 and PPARg^a^ at 0 and 24 h, respectively after injury. Muscles were harvested for analysis of the regenerative index 6 days after injury, and analysis of muscle function was performed 70 days after injury, (*n* = 8 mice per group). E) Quantification of the muscle regenerative index at 6 days and muscle fibrosis area at 70 days after injury treated with PGI2 and PPARg^a^ in young adult mice and aged mice, relative to DMSO vehicle control, (*n* = 8 mice per group). F) Assessment of relative bone mineral density in young and aged mice treated with DMSO vehicle control, or PGI2 and PPARg^a^ after injury (*n* = 8 mice per group). G) Total body weights (% of initial weights) of young mice and aged mice treated with DMSO, or PGI2 and PPARg^a^ after injury (*n* = 8 mice per group). H) Limb mass (% of body mass) of young mice and aged mice treated with DMSO, or PGI2 and PPARg^a^ after injury at the end of 70 days after injury (*n* = 8 mice per group). I) Assessment of the treadmill time to exhaustion for young mice and aged mice treated with DMSO, or PGI2 and PPARg^a^ after injury. * *p* < 0.05, ** *p* < 0.01(*n* = 8 mice per group). J) Assessment of grip strength in young and aged mice treated with DMSO vehicle control, or PGI2 and PPARg^a^ after injury (*n* = 8 mice per group). K) Model summarizing the effects of PGI2‐PPARg/PGC1a‐FAO signaling on the different phases of muscle regeneration in normal and aged mammals. Data were expressed as mean ± SEM. 2‐tailed Student‘s t‐test was used in E right and (J‐I), One‐way ANOVA with Bonferroni's post hoc test was used in C and E left. * *p* < 0.05, ** *p* < 0.01, *** *p* < 0.001.

We further postulated that PGI2‐induction of PPAR*γ* protein levels could require and synergize with PPAR*γ*/PGC1a activators to further enhance skeletal muscle regeneration in vivo. Given our findings on PGI2's induction of PPAR*α*/*γ* in myoblasts (Figure 4), we injected PGI2 at 0 h and PPAR‐specific modulators intramuscularly at 24 h after cryoinjury. Immunofluorescence analysis of eMHC+ myofibers revealed that neither the PPAR*α* agonist nor the PPAR*α* inhibitor had any significant effects compared to the PGI2 group (Figure S7D,E, Supporting Information). However, PGI2 synergized with the PPAR*γ* agonist to increase the muscle regenerative index, compared to PGI2 alone (**Figure** **8**A) or PPAR*γ* agonist alone (Figure 7). In contrast, the PPAR*γ* inhibitor blocked PGI2's effect on muscle regeneration, indicating that PPAR*γ* is necessary for PGI2's pro‐regenerative effect in vivo (Figure 8A–C).

While PPAR*γ* alone could enhance aged muscle regeneration, it was still far from young muscle in terms of function. Given the potency of the PGI2+PPAR*γ* spike combination in young muscles, and its deficiency and delay in aged muscles (Figures 2 and 6), we postulated that the spike combination might further enhance regeneration of aged muscles. Thus we analyzed the regenerative index in both young and aged mice 6 days after injury and administration of the PGI2‐PPAR*γ* spike combination (Figure 8D). Although aged mice had impaired muscle regeneration when compared to young adult mice (*p* < 0.001), the PGI2+PPAR*γ* agonist combination significantly improved the aged muscles’ regenerative index (*p* < 0.001, Figure 8E), and by a larger margin than PPAR*γ* agonist alone (Figure 7P).

To further investigate the long‐term functional effects of the PGI2+PPAR*γ* spike combination, we monitored the changes in body weight, endurance, and grip strength for 70 days after injury. Young mice’ body weight grew over time as expected, and the PGI2+PPAR*γ* spike combination had no significant effects on young healthy mice’ weights (Figure 8F). In contrast, aged mice’ body weight decreased over time as their muscles began to waste away with sarcopenia. The PGI2+PPAR*γ* spike combination slightly increased aged mice’ body weight (Figure 8F). Interestingly, the PGI2+PPAR*γ* spike combination significantly increased the limb muscle mass in both young and aged mice (Figure 8G). The PGI2+PPAR*γ* spike combination also increased their endurance time, especially for the aged mice, which approached young mice’ levels of endurance (Figure 8H). Moreover both young and aged mice’ grip strength were significantly increased (Figure 8I). These increases in muscle mass, endurance and strength were also accompanied by small but significant increases in bone mineral density for both young and aged mice (Figure 8J), indicating an overall improvement in exercise function.

Taken together, our experimental findings revealed that a transient spike of injury‐induced PGI2‐PPAR*γ*/PGC1a‐FAO during early myogenic stem cell differentiation can enhance and rejuvenate skeletal muscle regeneration, thus providing a novel therapeutic strategy for treating muscle diseases (Figure 8K).

## 3 Discussion

Myobolites are a class of metabolites induced by muscle injury or exercise, which might exert beneficial effects on muscular and overall health. While several myokine proteins have been found to partially explain the rejuvenative effects of physical exercise, it has remained unclear whether metabolites secreted by skeletal muscles could also play a similar role in promoting muscle function and general health. Here we used MSI‐based spatial metabolomics to reveal that several specific prostaglandin lipids and a prostacyclin in the injured regenerative ROI are injury‐induced myobolites. By scanning a small number of mice to find putative myobolites, then performing detailed biological experiments in vitro and in vivo, we were able to verify that the prostacyclin PGI2‐PPAR*γ*/PGC1a‐FAO axis is a key myobolite pathway. Our in‐depth validation results demonstrated that this pathway could be targeted to treat aged muscles.

While others have shown the importance of prostaglandins^[^ ^7^ ^]^, few have studied the mechanistic effects of prostacyclin on muscle stem cell differentiation during muscle regeneration. Our studies showed that the injury‐induced spike of prostacyclin PGI2 induced a spike of PPAR*γ*/PGC1a in pure MyoD+ myoblasts during early differentiation. The PPAR nuclear hormone receptors are metabolite‐responsive master regulators of lipid metabolism in many tissues.^[^ ^17^ ^]^ In particular, PPAR*γ* is known to be expressed and function not only in adipose tissues, but also in skeletal muscle.^[^ ^17^, ^18^ ^]^ However, previous studies with PPAR*γ* knockout or inhibition had led to controversy on the roles of PPAR*γ* in myogenesis.^[^ ^18^, ^19^ ^]^ Our results revealed there could be two possible reasons. Firstly, PPAR*γ* deficiency produced no overt phenotype in muscle development^[^ ^18^, ^19^ ^]^, because its long‐term deficiency leads to compensatory responses by other PPARs in myoblasts. Secondly, there is a critical difference between immortalized C2C12 cells and primary myoblasts, with regards to PPAR*γ* expression.^[^ ^20^ ^]^

Public data mining showed that immortalized C2C12 myoblasts already express high levels of PPAR*γ* (GDS586), and only downregulate PPAR*γ* upon terminal differentiation. In contrast to immortalized myoblasts, our work showed that primary myoblasts manifest a transient PPAR*γ* spike only during early commitment and differentiation. This has important implications for our interpretations of previous PPAR*γ*‐related data from immortalized C2C12 cells.

In collaboration with PGC1a and RXRg, both of which are increased by the PGI2 spike after muscle injury, the PPAR*γ*/PGC1a‐RXR transcription factor network can upregulate mitochondrial FAO.^[^ ^17c^ ^]^ FAO is emerging as an important metabolic pathway that has to be precisely controlled to regulate stem cell biology. Low levels of FAO are required for maintaining quiescent MuSCs, hematopoietic stem cells (HSCs), and intestinal stem cells (ISCs).^[^ ^21^ ^]^ However, excessive FAO can induce excessive mitochondrial ROS and stress signals to slow down cell growth and cause muscle degeneration during cachexia.^[^ ^15^ ^]^ But why does such a mechanism exist in our muscles in the first place? Our current study showed that an injury/inflammation‐induced FAO spike is normally needed for early differentiation of myoblasts during regeneration. The excessive inflammation‐FAO associated with aging leads to a delayed FAO spike and inhibited timely muscle regeneration. The downstream functions and mechanisms of FAO in cell fate regulation, including ROS signaling, lipid remodeling, acyl‐CoA oxidation, protein acetylation etc, are too diverse to list individually. But one possibility worth considering is that the surge of metabolites generated by long chain FAO, can in turn feedback as additional ligands for PPARs and act as a metabolic clock for the transient wave of PPAR‐transcription of mitochondrial and anti‐inflammatory genes^[^ ^22^ ^]^ during early muscle regeneration, before they are fully oxidized into CO_2_.

In general, we found that the spatio‐temporal regulation of lipid metabolism is surprisingly dynamic during muscle regeneration, with implications for the pharmacological restoration of endurance and strength in aged muscles. Thus, our study presents a case for novel drug combos that transiently activate the prostacyclin‐PPAR*γ*/PGC1a‐FAO axis to enhance muscle regeneration and exercise capacity, thereby ameliorating the degenerative effects of aging.^[^ ^23^ ^]^

## 4 Experimental Section

### Animals

Young (2 to 4 months) and old (24 to 29 months) wild‐type C57BL/6J mice were used in the studies. The mice were housed in cages under a 12‐h‐light–12‐h‐dark cycle. Animal rooms were maintained at ≈25 °C. Room lighting was automatically controlled on a 12‐h light, 12‐h dark schedule. All animal procedures were approved by the Institutional Animal Care and Use Committee (IACUC) of the Institute of Zoology (Chinese Academy of Sciences, IOZ‐IACUC‐2022‐170), and complied with all relevant ethical regulations.

### Matrix Application Setup

For each time point, three mice were sacrificed. Mice were anesthetized with isoflurane gas, decapitated with a guillotine, and their tibialis anterior (TA) muscles were removed and snap‐frozen in liquid nitrogen‐cooled isopentane. All muscle blocks were stored in a freezer at −80 °C until further preparation. All muscle blocks were cryosectioned using a Leica CM 1900 cryostat (Leica Microsystems LTD, Wetzlar, Germany). Serial muscle sections of 10 µm thickness were removed and thawed on ITO‐coated slides (Bruker Daltonik, Bremen, Germany) for positive ion mode MALDI MSI and polychromatic immunofluorescence analysis. Three consecutive muscle sections were taken from each sample as a group. The first and third sections were analyzed by immunofluorescence and Masson staining, and the second sections were analyzed by MSI. By comparing the Masson staining images to the MSI images, the damaged and undamaged regions could be accurately located and coregistered on SCiLS Lab Corea (Bruker Daltonik, Bremen, Germany) software. For MALDI MSI analysis, tissue sections were placed in a vacuum desiccator to dehydrate for 10 min at room temperature, before matrix was sprayed. A home‐built electric field‐assisted matrix coating setup was used to homogenously deposit matrix onto tissue sections. Briefly, 250 *µ*L of 100 mg mL^−1^ DHB matrix solution was deposited onto the tissue sections at a flow rate of 1 mL h^−1^. The automatic sprayer parameters for matrix coating were as follows: sheath gas flow, 0.1 MPa; heat temperature, 70 °C; reciprocating motion speed, 0.17 m s^−1^; high voltage between the sprayer needle and ITO slide, 5.3 kV; the distance between the spray needle and ITO slide, 12 cm.

In order to avoid batch effects, all sections in one‐time series group were imaged on a single ITO slide. Several slides were used for the time series in each figure, and different time point samples were randomly distributed among different slides with random ordering. The mouse muscle experiments were also started at T‐36h, T‐12h, etc, so that they all ended at the same time T, then the samples were harvested and frozen at the same time. The samples were also cryosectioned, randomly distributed on slides and imaged on the MALDI‐MSI in 1 batch, to further minimize batch effects.

### MALDI MSI analysis

MALDI ion source was used in all MSI experiments.FT‐ICR instrument (solariX 9.4T) equipped with a smartbeam II laser (Bruker Daltonik, Bremen, Germany) in the mass range of *m/z* 100–1000 in positive ion mode at a frequency of 1000 Hz, The spatial resolution for tissue samples was 100 µm, and the spatial resolution for standards calibration was 50 µm to establish the lower limits of detection (exact resolution noted in each figure). Bruker solariX’ Magnetic Resonance Mass Spectrometry (MRMS) data were initially visualized in FlexImaging 3.0 (Bruker), then imported into SCiLS Lab Core (Bruker Daltonik, Bremen, Germany) and coregistered with Masson staining images to identify normal and regenerative ROIs. Automatic import parameters were as follows: mass axis type was MRMS (Fourier‐transform), and average data point accuracy was 1.180 mDa. Peak finding and alignment were performed with the Move Peaks to Local Maxima tool, and used the following standard settings: ±0.156 Da *m/z* interval width, mean interval processing, and medium smoothing strength. Spatial denoising was performed with 3 × 3 neighborhoods. SCiLS Lab utilized the orthogonal matching pursuit algorithm to determine relevant peaks. This concept successively correlated a spectrum with different peaks at different positions and selects the most correlated peak. This peak was marked in the resulting *m/z* feature list and was removed from the spectrum before the procedure is reapplied. An *m/z* feature list for the resultant 2405 peaks (Table 1, Supporting Information) was exported through the report table function in SCiLS Lab Core. To obtain the average of the peak intensities within each region‐of‐interest (ROI), “Average Intensity” was selected under “Spot intensity statistics”. “Peak Area” was also selected under “Interval Processing Mode”, in order to measure each *m/z* peak as the centroided average of the peak area, which was the default mode for all data other than axial time‐of‐flight data according to the SCiLS Labs manual, which included the Bruker solariX MRMS data. MSI data were normalized using the root mean square (RMS) method to obtain normalized average intensity values for each *m/z*. Log_10_‐transformed (Normalised Average Intensity) values of each sample were found to be suitable for statistical testing. To confirm these quantitation results (represented as color images), the raw data were also processed with FlexImaging 3.0, with the data normalized using the RMS method and the Ymean/Ymax threshold set at 0.5 to reduce noise spectra, before exporting as grayscale images for visual and quantitative confirmation with ImageJ (NIH). The experimental *m/z* values were compared with the Human Metabolome Database (HMDB 5.0, www.hmdb.ca) and METLIN database (https://metlin.scripps.edu) using exact molecular weights and a maximum mass tolerance of 3 ppm.

### Titrations of Standards on ITO Slides and Tissue Sections for Calibration

All prostanoid standards were obtained from Cayman. PGI2, PGG2 PGD2 PGD1, and PGF1a stocks were dissolved to 3.25 mM in 80% methanol. PGE3, PGD1, PGJ2, and PGG2 stocks were dissolved to 1.4 mm, 14 mm, 15 mm, and 271 µm, respectively in 80% methanol, then titrated to 1000 times dilution. The tissue sections were placed in a vacuum desiccator, dehydrated at room temperature for 10 min, and 0.4 µL of the diluted standard was titrated onto the tissue sections and ITO slides around the tissue sections, then matrix was sprayed, and data were collected as described above.

### RNA Profiling

Total RNA was extracted using Trizol (Thermo Fisher) and reverse transcribed into cDNA with Superscript III (Thermo Fisher) according to manufacturer's instructions. The synthesized cDNA was diluted 5× in H_2_O before performing Qpcr with KAPA SYBR FAST (Merck) on ABI Prism 7900HT (Applied Biosystems) real‐time PCR system according to manufacturers’ instructions. Primer sequences are provided in Table S1 (Supporting Information). Total RNA was also further purified by ethanol precipitation and sent for mRNA sequencing. Paired‐end libraries were constructed and sequenced using an Illumina Nova‐PE150 platform at Novogene (Beijing, China). Further analysis was performed with Gene Set Enrichment Analysis (GSEA; Broad Institute).

### LC–MS/MS Metabolomics Analysis

Liquid chromatography–tandem mass spectrometry (LC‐MS/MS) metabolomics analyses were performed on equal numbers of cells according to previously published protocols.^[^ ^15^ ^]^ Briefly, an Acquity I‐class ultra‐performance liquid chromatography (UPLC) system coupled in line with a Xevo G2‐XS high‐resolution quadrupole‐time of flight hybrid mass spectrometer (Waters) was used. 25mM of [1,2‐^13^C]‐glucose (Cambridge Isotopes) was used to completely replace D‐glucose in DMEM for the ^13^C‐labeling flux kinetics study. 30 mm [U‐^13^C]‐palmitate was conjugated to fatty acid‐free BSA to replace albumin in the KnockOut Serum Replacement. The extraction procedure and PGI2 quantitative analysis by LC–MS/MS were based on previously described methods.^[^ ^24^ ^]^ Briefly, young and aged muscles were harvested, weighed, and snap‐frozen with liquid nitrogen. Muscle tissue was homogenized with homogenization buffer (acetone/water 1:1 v/v; 0.005% BHT to prevent oxidation), followed by centrifugation and supernatant collection. Equal volumes of hexane were added to the samples, then vortexed, centrifuged, and frozen at −80 °C. The frozen lower aqueous layer was collected and mix with 25 µL of 1 m formic acid. Equal chloroform was added to the aqueous phase and shaken for 15 min to ensure full extraction. After centrifugation, the lower chloroform layer was collected and evaporated to dryness under nitrogen at 40 °C. The dry residue was reconstituted in acetonitrile/10 mm ammonium acetate (2:8 v/v) and analyzed by LC–MS/MS.

### Cell Culture and Drug Treatment

Primary human skeletal muscle (HSKM) myoblasts were isolated by FACS for CD82^+^ CD31^−^ CD45^−^ Lin^−^ cells, and cultured on gelatin (0.1%, Merck‐Millipore)‐coated plates under a humidified atmosphere (5% CO_2_ and 37 °C) with growth medium composed of DMEM/F‐12 (Gibco) supplemented with fetal bovine serum (FBS) (20%, GE Healthcare), l‐glutamine (1%, Gibco), and penicillin‐streptomycin (1%, Gibco). Primary HSKM myoblasts were validated to be 100% MyoD+ by immunofluorescence.^[^ ^16^ ^]^ Confluent HSKM myoblasts were induced to differentiate by replacing growth media with differentiation medium, comprising of DMEM/F‐12, KnockOut Serum Replacement (1%, Gibco), l‐glutamine (1%, Gibco), and penicillin‐streptomycin (1%, Gibco). For drug treatments, cells were incubated with etomoxir (5 µm, Cayman Chemical), rosiglitazone (10 µm, Merck), GW9662 (0.1 µm, Cayman Chemical). MitoTracker Red (200 nm, Thermo Fisher), and JC1 (2 µm, Thermo Fisher) staining were performed according to manufacturer's instructions and stained cells were imaged with a Zeiss fluorescence microscope.

### Mitochondrial DNA Copy Number Measurement by qPCR

After HSKM myoblasts were induced to differentiate, HSKM cells were harvested every 12 h. Genomic DNA was isolated from HSKM cells using the DNeasy Blood & Tissue Kit (Qiagen) according to manufacturer's instructions. Briefly, HSKM cells were washed with phosphate buffered saline (PBS) (Thermo Fisher), trypsinized (0.25%, Thermo Fisher) at 37 °C for 3 min and centrifuged at 1300 rpm for 3 min. The harvested cell pellets were subsequently stored at −80 °C, until all the samples were available for DNA isolation. For mitochondrial DNA copy number measurement, qPCR‐based mitochondrial quantification was performed using KAPA SYBR FAST (Merck) on ABI Prism 7900HT (Applied Biosystems) real‐time PCR system according to manufacturers’ instructions. Primer sequences are provided in Table S2 (Supporting Information).

### Oxygen Consumption Analysis

HSKM myoblasts were seeded onto Seahorse XF96 Cell Culture Microplate (Agilent), pre‐coated with gelatin (0.1%, Merck‐Millipore), in growth media at 10 000 cells per well. Two days after seeding, HSKM myoblasts were induced to differentiate by replacing growth media with differentiation medium. Before performing the Seahorse XF cell Mito Stress Test assay, cell culture media were replaced with assay media containing only palmitate‐conjugated BSA (Agilent) and incubated in a CO_2_‐free incubator at 37 °C for 1 h to equilibrate temperature and pH for each well. During the assay, Oligomycin (2 µm, Agilent), FCCP (0.5 µm, Agilent), and a mixture of Antimycin A and Rotenone (0.5 µm, Agilent), were injected sequentially and measurements were taken according to manufacturer's instructions. The data were analyzed using WAVE software.

### Western Blot Analysis

Protein was extracted with RIPA buffer (Thermo Fisher) supplemented with protease inhibitor cocktails I and II (Merck) and phosphatase inhibitor cocktail set III (Merck). Protein was quantified with Pierce BCA protein assay kit (Thermo Fisher) and analysed with Sunrise Tecan plate reader. After SDS‐PAGE and electro‐transfer onto PVDF membranes (GE Healthcare), western blot was performed with the following primary antibodies, Pax7 (1:500,AB_528428, Developmental Studies Hybridoma Bank), MyoD (1:400, sc37746, Santa Cruz Biotechnology), myogenin (1:400, sc‐52903, Santa Cruz Biotechnology), myosin heavy chain eFluor 660 (1:20, 50‐6503‐82, Thermo Fisher), *α*‐actinin (Sarcomeric) (1:500, A7811, Merck), PPARg (1:400, sc‐7273, Santa Cruz Biotechnology), PPARa (1:400, sc‐398394, Santa Cruz Biotechnology), PGC1a (1:400, sc‐518025, Santa Cruz Biotechnology), MYH3(1:400,sc‐53091, Santa Cruz Biotechnology), Rxrg (1:400, sc‐514134, Santa Cruz Biotechnology), CoxIV (1:1000,ab33985,abcam), Cytochrome C (1:1000, ab133504, abcam), Cpt1b (0.2µg/ml, PA5‐79065, ThermoFisher Scientific), Prostaglandin I2 Synthase (1:1000, NBP1‐62390, Novus Biologicals), PTGIR (1:1000, NBP2‐93654, Novus Biologicals) and GAPDH (1:1000, sc‐25778, Santa Cruz Biotechnology). Subsequently, blots were stained with secondary antibody anti‐rabbit IgG HRP conjugate (1:2500, W401B, Promega) and anti‐mouse IgG HRP conjugate (1:2500, W402B, Promega). Protein levels were detected using ECL prime western blotting detection reagent kit (GE Healthcare).

### Immunofluorescence Analysis

Cells were washed with PBS (Thermo Fisher) and fixed with paraformaldehyde (PFA) (4%, Electron Microscopy Sciences) at room temperature for 10 min. Cells were stained with primary antibody myosin heavy chain eFluor 660 (1:20, 50‐6503‐82, Thermo Fisher) at 4 °C overnight. DAPI (Merck) was used as a nuclear counterstain according to manufacturer's recommendations. Stained cells were imaged with a Zeiss fluorescence microscope. Immunofluorescence analysis of tissue sections was performed with primary antibodies against MHC I (BA‐D5), MHC IIa (SC‐71), and MHC IIb (BF‐F3). All these primary antibodies were purchased from the Developmental Studies Hybridoma Bank (University of Iowa). For signal detection, Alexa Fluor 488, 594, or 647‐conjugated secondary antibodies (Invitrogen, 1:100) were used.

### RNA Transfection and shRNA Transduction of Cells

HSKM myoblasts were seeded onto gelatin‐coated plates and cultured in growth media. One day after seeding, a mixture of polyethylenimine (PEI)/RNA was used to transfect the HSKM cells. To prepare the mixtures, PEI together with the following RNAs were mixed with serum‐free DMEM, hsa‐let‐7 miRCURY LNA microRNA Power Family Inhibitor (YFI0450006, Qiagen), a combination of mirVana miRNA mimic hsa‐let‐7a‐5p (4464066, Assay ID: MC10050, Thermo Fisher) and mirVana miRNA mimic hsa‐let‐7b‐5p (4464066, Assay ID: MC11050, Thermo Fisher), MYOD1 siRNA (4392420, siRNA ID: s9231, Thermo Fisher), or Cy5‐conjugated scramble RNAi control. The PEI/RNA transfection mixtures were incubated at room temperature for 20 min before being added to the HSKM cells. The transfection media was replaced with growth media after 24 h. Mission shRNA sequences (Sigma) were cloned into a lentiviral TetOff‐shRNA‐GFP vector (RRID: Addgene_11779) and then transfected into 293FT HEK cells (Clontech) seeded at 10% confluency via a 42 µL: 7 µg: 6.3 µg: 0.7 µg mix of PEI (1 mg mL^−1^): lentiviral plasmid: dR8.2 packaging plasmid (RRID: Addgene_8455): VSV‐G envelope plasmid (RRID: Addgene_8454). Lentiviral supernatants were collected within the 48‐ to 96‐h window and filtered with a 0.45 µm filter (Sartorius). Lentiviral supernatants were pooled and concentrated by spinning through 100 000 NMWL Amicon Ultra centrifugal filters (Merck Millipore) at 4000 × *g*, at 4 °C for 15 min. Lentiviral shRNA transduction of HSKM myoblasts was performed for 48 h, followed by FACS for GFP+ cells.

### Skeletal Muscle Cryoinjury

Mice were anesthetized with a mixture of ketamine and xylazine (120 and 8 mg kg^−1^, respectively) via intraperitoneal injection. After successful anesthetization, the skin over the tibialis anterior (TA) muscle was disinfected by wiping with 70% ethanol, and a 3mm incision was made over the TA muscle. A dry‐ice‐chilled 4‐mm metal probe was directly applied onto the exposed TA muscle for three cycles of five seconds to induce cryoinjury. Thereafter, the incision was immediately sutured using a surgical suture stapler. Upon recovery under heat lamps for a period of 2 h, the mice were randomly allocated to each treatment groups. All the drugs (PGs (13 nmol, Cayman), rosiglitazone (20 mg kg^−1^, Merck) and etomoxir (20 mg kg^−1^, Merck)) and vehicle were intramuscularly injected into the muscle using an insulin syringe (BD). Mouse grip strength was measured as the average of six measurements of the maximal peak force generated on a grip strength meter (Bioseb). Mouse endurance was performed time to exhaustion by treadmill, the speed of which was increased every 2 min by 5 cm s^−1^ with the slope at 13% (up). Mice were considered to be exhausted when the animal's hindlimbs remained on the electric grid for more than 10s. TA muscles were harvested for histology, immunofluorescenc,e and western blots. For the histology samples, TA muscles were incubated in 4% PFA solution overnight and embedded in paraffin. Samples were serially sectioned until depleted and haemotoxylin and eosin (H&E) or Masson trichrome staining were performed on every 12th 5‐µm‐thick tissue section. After microscopy imaging, the area of the cryoinjured myofibers was quantified using ImageJ. For western blot samples, TA muscles were snap‐frozen in liquid nitrogen and homogenized in RIPA buffer (Thermo Fisher) supplemented with protease inhibitor cocktails I and II (Merck) and phosphatase inhibitor cocktail set III (Merck) using TissueLyser II (Qiagen).

### Intramuscular Injection of GFP‐Positive HSKM Cells

Lentiviral eGFP expression vector pLenti CMV GFP (Addgene #17445) was packaged into lentiviral particles. To obtain GFP‐positive HSKM myoblasts, cells were then transduced with the viral particles and selected with growth media containing blasticidin (25 µg/ml, InvivoGen) for 5–7 days. Cryoinjury was carried out on eight‐week‐old NSG mice as mentioned above and subsequently mice were randomly allocated into TWO groups for HSKM transplantation, rosiglitazone‐treated GFP‐positive HSKM, and DMSO‐treated GFP‐positive HSKM. GFP‐positive HSKM were treated with growth media containing rosiglitazone or DMSO control for 24 h and trypsinized for cell transplantation. Two million HSKM cells were resuspended in 100 *µ*L of growth media containing Matrigel hESC‐Qualified Matrix (1:1, Corning). Using a 23‐gauge needle, the cell suspension was injected into the TA muscle. Four days after cryoinjury, the TA muscles were harvested in 4% PFA overnight and embedded in paraffin.

### Immunohistochemistry

TA tissue samples embedded in paraffin were sectioned using a microtome and transferred onto Leica Microsystems Plus Slides. Paraffin‐embedded sections were deparaffinized in xylene (Merck) for two washes (10 min) and then transferred sequentially into 100% EtOH (Merck), 100% EtOH, 95% EtOH, and 70% EtOH (2 min) at room temperature. The sections were then rehydrated in deionized water (3mins). Antigen retrieval was carried out using the 2100 Retriever in sodium citrate buffer (Merck, pH 6.2, 30 min). Slides were then cooled in cold PBS (15 min) and blocked‐in blocking buffer at room temperature (30mins). Primary antibody staining was conducted in blocking buffer at 4 °C overnight with the following antibodies, MyoD (1:40, sc‐377460, Santa Cruz Biotechnology), Pax7 (1:6, AB528428, Developmental Studies Hybridoma Bank), MYH3 (1:40, sc‐53091 Santa Cruz Biotechnology), Laminin (1:100, PA1‐16730, Invitrogen), F4/80 (1:100, AB6640, Abcam), PDGFRa (1:200, ab203491, Abcam), Ki67 (1:200, 14‐5698‐82, Invitrogen), GFP (1:500, sc‐9996, Santa Cruz Biotechnology), and myosin heavy chain eFluor 660 (1:20, 50‐6503‐82, eBioscience). After the slides were washed thrice in PBS (10 min) and counterstained with DAPI, secondary antibody staining for GFP was conducted in blocking buffer at room temperature (1 h) with goat anti‐mouse IgG secondary antibody, Alexa Fluor 488 (1:500, A11001, Thermo Fisher).

### Gene Expression Omnibus Database

Transcriptomic datasets were mined on immortalized C2C12 cells (GDS586) and primary human myoblast differentiation (GSE55034) in the Gene Expression Omnibus (GEO) database.

### Statistical Analysis

Pre‐processing of data^[^ ^25^ ^]^ was performed with either FlexImaging, SCiLS Lab Core, Excel, GraphPad Prism, or the R Bioconductor package. Sample sizes (n) and statistical methods used to assess significant differences in each statistical analysis are noted in each figure legend. All data were expressed as mean ± SEM (standard error of mean). The normal distribution of samples was determined by the Shapiro–Wilk normality test, and the equality of sample variances was determined by the Levene's Homogeneity of Variance test.^[^ ^26^ ^]^ When both the Shapiro–Wilk test (*p* > 0.05 for both samples) and the Levene's test (*p* > 0.05) were passed, the parametric 2‐tailed Student's test was used to analyze the difference between the two groups; otherwise, the non‐parametric Mann‐Whitney *U* test (also known as the Wilcoxon rank‐sum test) was used.^[^ ^26^ ^]^ One‐way analysis of variance (ANOVA) was used for statistical analysis of multigroup comparisons, and Bonferroni post hoc tests were used to assess the significance of the differences between the two groups. Log_10_‐transformed (normalized average intensities) were used.^[^ ^26^ ^]^ For all experiments, *p* <  0.05 was considered to be significant and represented by “#” or “*”; *p* <  0.01 was represented by “**”; *p* <  0.001 was represented by “***”. SPSS 24.0 was used for the above statistical analyses. Graphing was performed in GraphPad Prism software (version 6.01) and Excel.

## Conflict of Interest

The authors declare no conflict of interest.

## Author Contributions

L.L., Y.‐J.B.C., T.L., and K.L. contributed equally to this work. L.L., Y.‐J.B.C., T.L., K.L., and N. S.‐C. designed all experiments. L.L., K.L., T.L., Y.‐J.B.C., M.‐W. J.C., W.M., J.‐W.G., Y.W., J.S., and C.L. performed in vitro and in vivo experiments and collected the data. M.‐W. J.C. designed, performed, and analyzed in vivo engraftment studies. K.L. and Y.‐J.B.C. purified and maintained primary myoblasts. B.T.T. and K.Y. provided muscle samples. T.L., L.L., K.L., W.M., and K.H.L. designed, performed, and analyzed MALDI MSI. Y.S.H. helped in the design and analysis of LC–MS/MS metabolomics. All authors analyzed the data and wrote the manuscript.

## Supporting information

Supporting Information

Supplemental Table 1

Supplemental Table 2

Supplemental Table 3

## Data Availability

The data that support the findings of this study are available in the supplementary material of this article.
