# Supplementary material for: Muscle Injuries Induce a Prostacyclin‐PPARγ/PGC1a‐FAO Spike That Boosts Regeneration
Source: Adv Sci (Weinh). 2023 May 4;10(21):2301519. doi: 10.1002/advs.202301519 (PMC10375192; doi:10.1002/advs.202301519)
Supplement: Supplementary file 1 — Supporting Information [file ADVS-10-2301519-s003.pdf]

ADVANCED SCIENCE
Open Access

Supporting Information

for *Adv. Sci.*, DOI 10.1002/advs.202301519

Muscle Injuries Induce a Prostacyclin-PPARγ/PGC1a-FAO Spike That Boosts Regeneration

*Lanfang Luo, Yan-Jiang Benjamin Chua, Taoyan Liu, Kun Liang, Min-Wen Jason Chua, Wenwu Ma, Jun-Wei Goh, Yuefan Wang, Jiali Su, Ying Swan Ho, Chun-Wei Li, Ke Hui Liu, Bin Tean Teh, Kang Yu and Ng Shyh-Chang**

Figure S1.

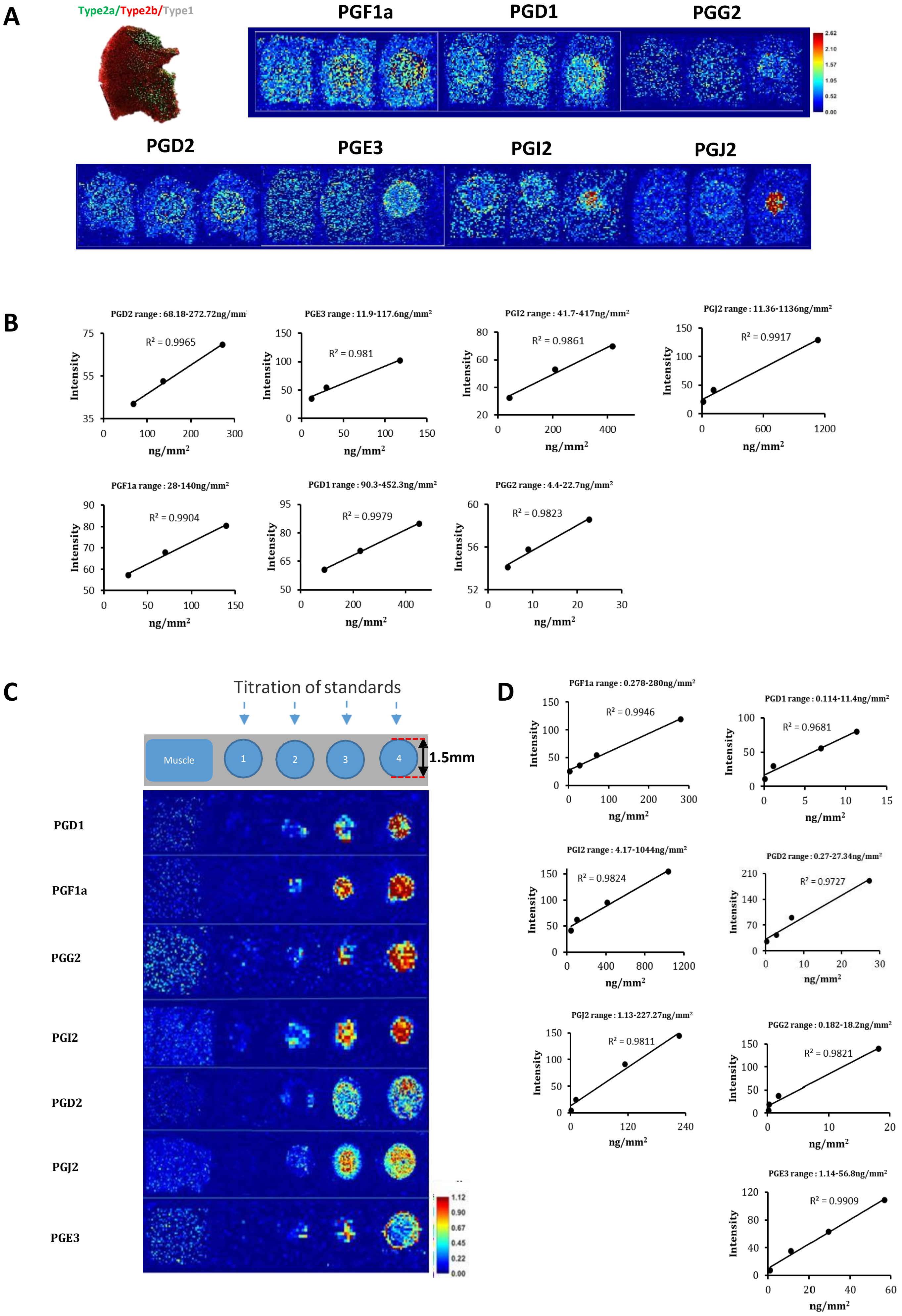

A
Type2a/Type2b/Type1
PGF1a
PGD1
PGG2
PGD2
PGE3
PGI2
PGJ2
2.62
2.10
1.57
1.05
0.52
0.00
B
PGD2 range : 68.18-272.72ng/mm
R² = 0.9965
PGE3 range : 11.9-117.6ng/mm²
R² = 0.981
PGI2 range : 41.7-417ng/mm²
R² = 0.9861
PGJ2 range : 11.36-1136ng/mm²
R² = 0.9917
PGF1a range : 28-140ng/mm²
R² = 0.9904
PGD1 range : 90.3-452.3ng/mm²
R² = 0.9979
PGG2 range : 4.4-22.7ng/mm²
R² = 0.9823
Intensity
ng/mm²
C
Titration of standards
Muscle
1
2
3
4
1.5mm
PGD1
PGF1a
PGG2
PGI2
PGD2
PGJ2
PGE3
1.12
0.90
0.67
0.45
0.22
0.00
D
PGF1a range : 0.278-280ng/mm²
R² = 0.9946
PGD1 range : 0.114-11.4ng/mm²
R² = 0.9681
PGI2 range : 4.17-1044ng/mm²
R² = 0.9824
PGD2 range : 0.27-27.34ng/mm²
R² = 0.9727
PGJ2 range : 1.13-227.27ng/mm²
R² = 0.9811
PGG2 range : 0.182-18.2ng/mm²
R² = 0.9821
PGE3 range : 1.14-56.8ng/mm²
R² = 0.9909
Intensity
ng/mm²

FIG. S1. Titrations of several prostanoids to confirm the accuracy and dynamic range of detection by MALDI MSI.

(A) Top left, multicolor immunofluorescence staining of the TA muscle cryosection. Type2a, green; Tpye2b, red; TypeI, white. Different concentrations of prostanoid droplets were titrated onto cryosections of the TA muscle tissue, and subjected to MALDI MSI (80 x 80 μm pixel).

(B) Standard curves of prostanoids titrated onto the tissue section (80 x 80 μm pixel), Data were expressed as mean gray value.

(C) MSI analysis of different doses of prostanoid standards, compared to TA muscle cryosections, on ITO slides (50 x 50 μm pixel).

(D) Standard curves of prostanoids titrated onto ITO slides. (50 x 50 μm pixel), Data were expressed as mean gray value.

Figure S2.

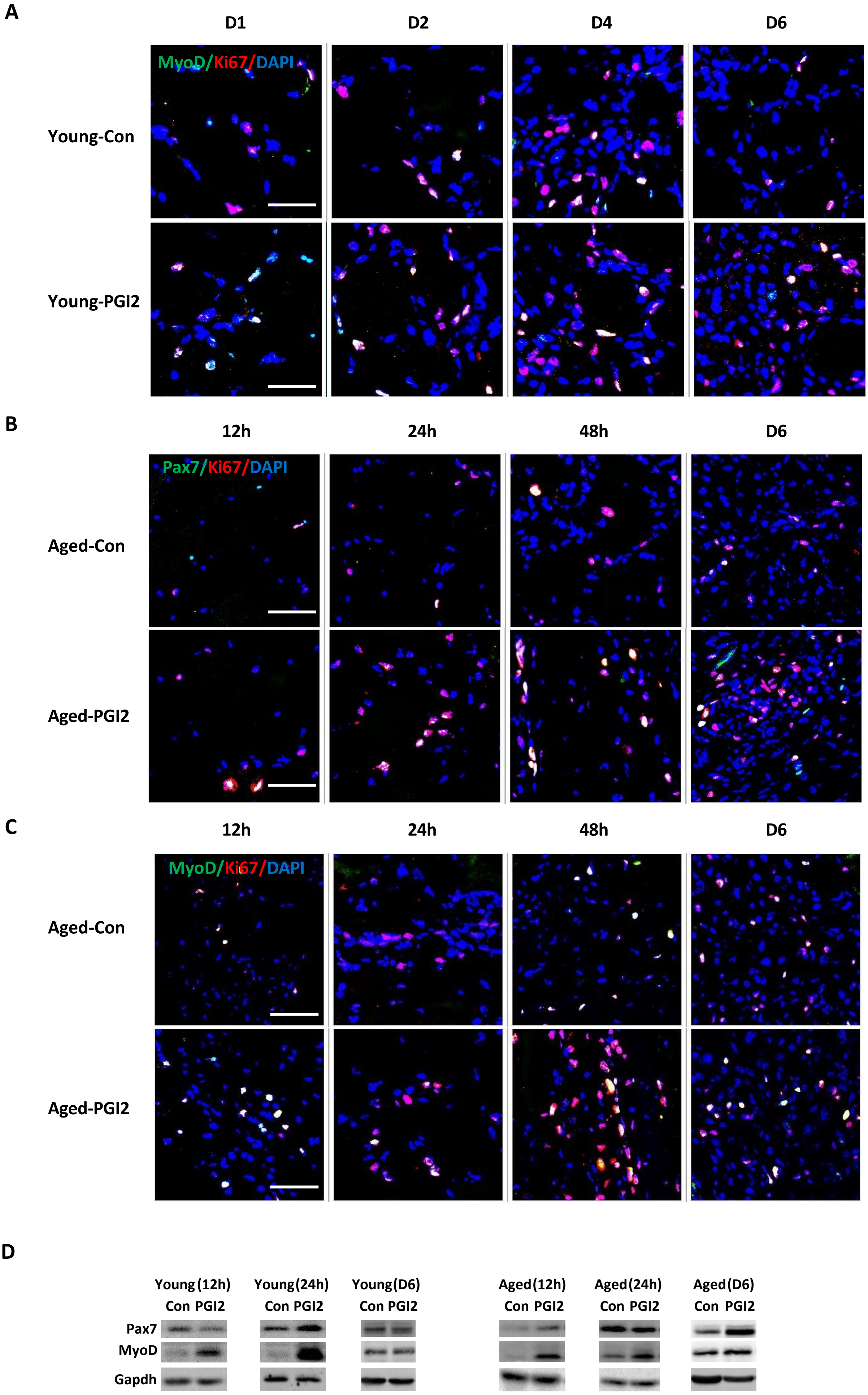
A
D1
D2
D4
D6
MyoD/Ki67/DAPI
Young-Con
Young-PGI2
B
12h
24h
48h
D6
Pax7/Ki67/DAPI
Aged-Con
Aged-PGI2
C
12h
24h
48h
D6
MyoD/Ki67/DAPI
Aged-Con
Aged-PGI2
D
Young (12h)
Young (24h)
Young (D6)
Aged (12h)
Aged (24h)
Aged (D6)
Con PGI2
Con PGI2
Con PGI2
Con PGI2
Con PGI2
Con PGI2
Pax7
MyoD
Gapdh

FIG. S2. PGI2 upregulates Pax7+ muscle stem cells and MyoD+ myoblasts in young and aged mice .

(A) Representative immunofluorescence images.Young mice(N=3). MyoD+ myoblasts and Ki67+ proliferative cells at different time-points of regeneration after cryoinjury of the TA muscle in young mice, followed by intramuscular injection of PGI2 or the vehicle control (Con); MyoD, green; Ki67, red;DAPI, blue;Scale bar, 50μm.

(B) Representative immunofluorescence images.Aged mice(N=3). Pax7+ muscle stem cells and Ki67+ proliferative cells at different time-points of regeneration after cryoinjury of the TA muscle in aged mice, followed by intramuscular injection of PGI2 or the vehicle control (Con). Pax7, green; Ki67, red; DAPI, blue; Scale bar, 50μm .

(C) Representative immunofluorescence images.Aged mice(N=3). MyoD+ myoblasts and Ki67+ proliferative cells at different time-points of regeneration after cryoinjury of the TA muscle in aged mice, followed by intramuscular injection of PGI2 or the vehicle control (Con); MyoD, green; Ki67, red. DAPI, blue; Scale bar, 50μm .

(D) Representative immunofluorescence images.Western blots for Pax7 and MyoD protein expression in young(N=3) and aged(N=3) muscles (12h, 24h or 6 days post-injury) after injection of PGI2 post-injury, relative to the vehicle control (Con).

Figure S3.

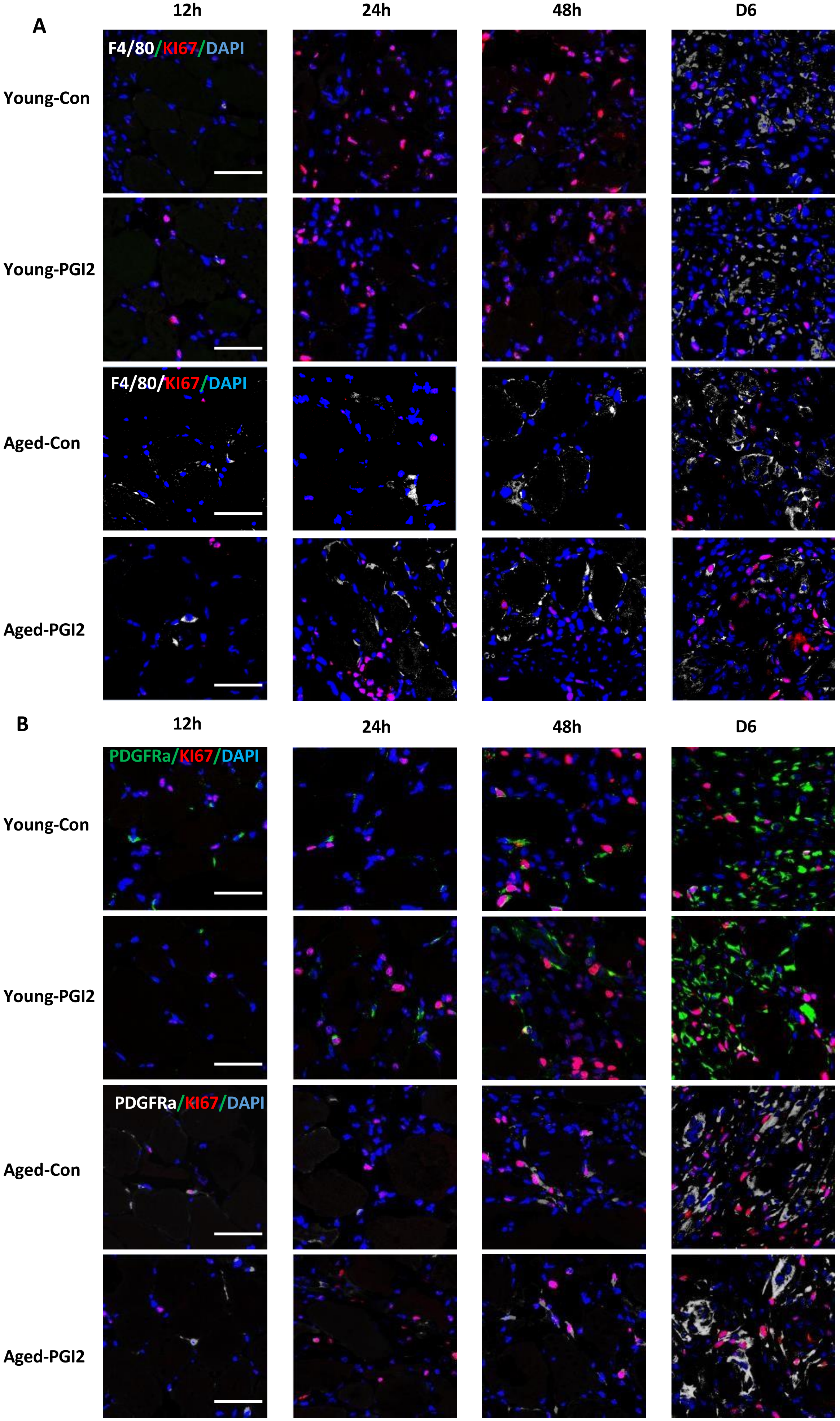
A
12h
24h
48h
D6
F4/80/KI67/DAPI
Young-Con
Young-PGI2
F4/80/KI67/DAPI
Aged-Con
Aged-PGI2
B
12h
24h
48h
D6
PDGFRa/KI67/DAPI
Young-Con
Young-PGI2
PDGFRa/KI67/DAPI
Aged-Con
Aged-PGI2

FIG. S3. Macrophages and FAPs are not affected by PGI2 in young and aged mice.

(A) Representative immunofluorescence images. Macrophages (F4/80+) at different time-points of regeneration after cryoinjury of the TA muscle in young(N=3) mice (top panel) and aged(N=3) mice (bottom panel), after intramuscular injection of PGI2 or the vehicle control (Con). F4/80, white; Ki67, red; DAPI, blue; Scale bar, 50μm .

(B) Representative immunofluorescence images. FAPs (fibro-adipogenic precursors, PDGFRa+) at different time-points of regeneration after cryoinjury of the TA muscle in young (N=3) mice (top panel) and aged (N=3) mice (bottom panel), after intramuscular injection of PGI2 or the vehicle control (Con,N=3)). Young muscle: PDGFRa, green; Ki67, red; Aged muscle: PDGFRa, white; Ki67, red; DAPI, blue. Scale bar, 50μm

Figure S4.

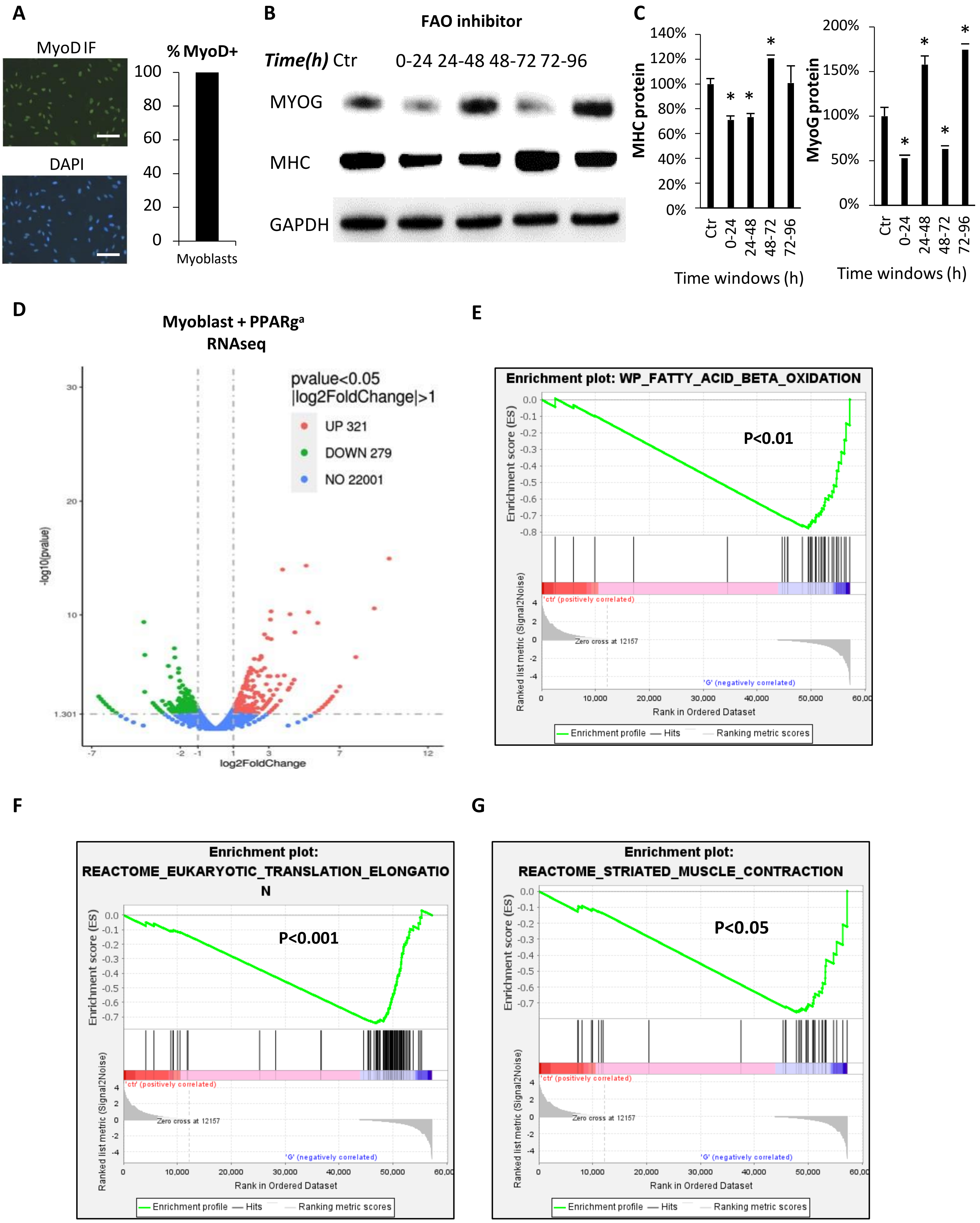

A
MyoD IF
DAPI
% MyoD+
100
80
60
40
20
0
Myoblasts
B
FAO inhibitor
Time(h) Ctr 0-24 24-48 48-72 72-96
MYOG
MHC
GAPDH
C
MHC protein
MyoG protein
Time windows (h)
Ctr
0-24
24-48
48-72
72-96
D
Myoblast + PPARg[a]
RNAseq
pvalue<0.05
|log2FoldChange|>1
UP 321
DOWN 279
NO 22001
-log10(pvalue)
log2FoldChange
E
Enrichment plot: WP_FATTY_ACID_BETA_OXIDATION
P<0.01
F
Enrichment plot:
REACTOME_EUKARYOTIC_TRANSLATION_ELONGATION
P<0.001
G
Enrichment plot:
REACTOME_STRIATED_MUSCLE_CONTRACTION
P<0.05
Enrichment score (ES)
Ranked list metric (Signal2Noise)
'ctr' (positively correlated)
'G' (negatively correlated)
Zero cross at 12157
Rank in Ordered Dataset
Enrichment profile
Hits
Ranking metric scores

FIG. S4. PPAR-FAO is necessary and sufficient to control myogenesis

(A) Immunofluorescence (IF) analysis of MyoD+(green) myoblasts in culture. Nuclei were counterstained with DAPI(blue). Scale bar, 50μm (N=3).

(B) Western blot analysis of MyoG and MHC, relative to GAPDH, in myocytes after treatment with the CPT1 inhibitor etomoxir (5μM) during different time-windows in myogenic differentiation.

(C) Quantification of MHC protein levels (left panel) and MyoG protein levels (right panel) in myocytes after treatment with the CPT1 inhibitor etomoxir (5μM) during different time-windows in myogenic differentiation (N=3).

(D) Volcano plot of genes that were differentially expressed in myocytes after treatment with 10μM of the PPARg agonist rosiglitazone for 48h(N=3).

(E) The fatty acid oxidation (FAO) gene signature was significantly upregulated in myocytes after treatment with 10μM of PPARg agonist rosiglitazone for 48h (N=3). * P<0.05, ** P<0.01, *** P<0.001.

(F) The eukaryotic ribosomal translation elongation signature, associated with muscle growth, was significantly upregulated in early myocytes after treatment with 10μM of the PPARg agonist rosiglitazone (PPARg^{a}, (N=3)).

(G) The striated muscle (or myogenesis) signature was significantly upregulated in myocytes after treatment with 10μM of PPARg agonist rosiglitazone (PPARg^{a}, (N=3)).

Data were expressed as mean ± SEM. 2-tailed Student's t-test was used in C.

Figure S5.

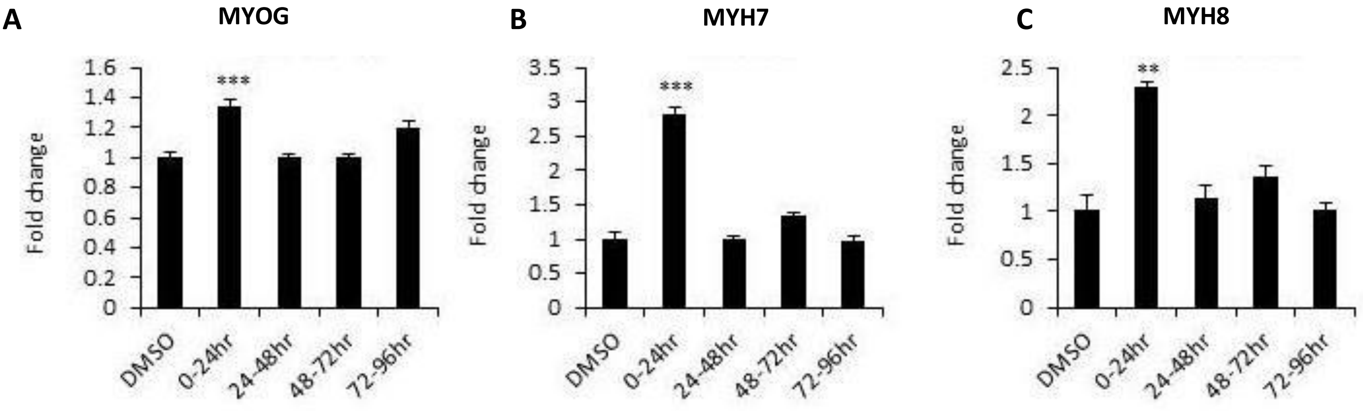

FIG. S5. Only PPARg activation at the early differentiation phase promotes myogenesis.

(A) Quantitative PCR for MYOG expression in myocytes, after treatment of the cells with the PPARg agonist rosiglitazone at different time-windows of myogenic differentiation.

(B) Quantitative PCR for MYH7 expression in myocytes, after treatment of the cells with the PPARg agonist rosiglitazone at different time-windows of myogenic differentiation.

(C) Quantitative PCR for MYH8 expression in myocytes, after treatment of the cells with the PPARg agonist rosiglitazone at different time-windows of myogenic differentiation.

Data were expressed as mean ± SEM. 2-tailed Student's t-test was used in (A-C). ** $P < 0.01$, *** $P < 0.001$, N=3 biological replicates unless mentioned otherwise.

Figure S6.

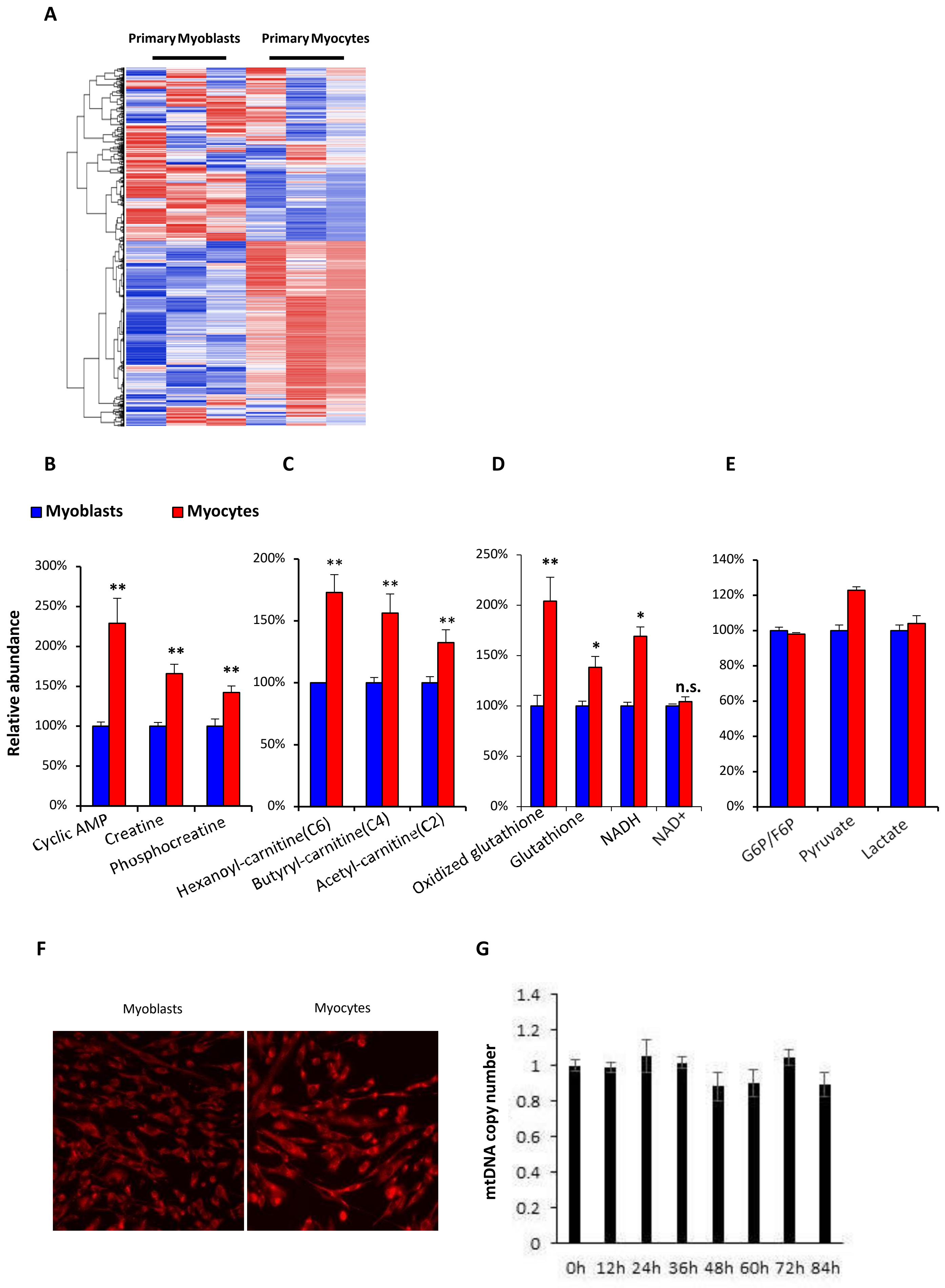

A
Primary Myoblasts
Primary Myocytes
B
C
D
E
Myoblasts
Myocytes
Relative abundance
300%
250%
200%
150%
100%
50%
0%
**
**
**
Cyclic AMP
Creatine
Phosphocreatine
200%
150%
100%
50%
0%
**
**
**
Hexanoyl-carnitine(C6)
Butyryl-carnitine(C4)
Acetyl-carnitine(C2)
250%
200%
150%
100%
50%
0%
**
*
*
n.s.
Oxidized glutathione
Glutathione
NADH
NAD+
140%
120%
100%
80%
60%
40%
20%
0%
G6P/F6P
Pyruvate
Lactate
F
Myoblasts
Myocytes
G
mtDNA copy number
1.4
1.2
1
0.8
0.6
0.4
0.2
0
0h
12h
24h
36h
48h
60h
72h
84h

FIG. S6. Mitochondrial FAO involved in early myoblast differentiation.

(A) Clustergram heatmap of intracellular metabolites, as measured by LC-MS/MS, in post-mitotic, mononucleated, primary human myocytes after differentiation for 48h, relative to primary human myoblasts isolated by FACS.

(B) Relative abundance of metabolites that serve as hallmarks of myogenic differentiation: cyclic AMP (cAMP), creatine and phosphocreatine.

(C) Relative abundance of acyl-carnitines, ranging from the 2-carbon (C2) acetyl-carnitine to the 6-carbon (C6) hexanoyl-carnitine.

(D) Relative abundance of metabolites that regulate the redox balance, including both the oxidized and reduced forms of glutathione and nicotinamide adenine dinucleotide (NAD+ and NADH).

(E) Relative abundance of key glycolytic intermediates, glucose-6-phosphate (G6P) or fructose-6-phosphate (F6P), pyruvate and lactate.

(F) Mitochondrial volume in post-mitotic mononucleated human myocytes after differentiation for 48h, relative to undifferentiated proliferative myoblasts, by fluorescence staining with Mitotracker Red. Scale bar, 50μm.

(G) Quantification of relative mitochondrial DNA (mtDNA) copy numbers in primary human myoblasts undergoing differentiation for 84h.

Data were expressed as mean ± SEM. 2-tailed Student's t-test was used in (B-E) and G
*<0.05,** P < 0.01, *** P < 0.001, N=3 biological replicates unless mentioned otherwise.

Figure S7.

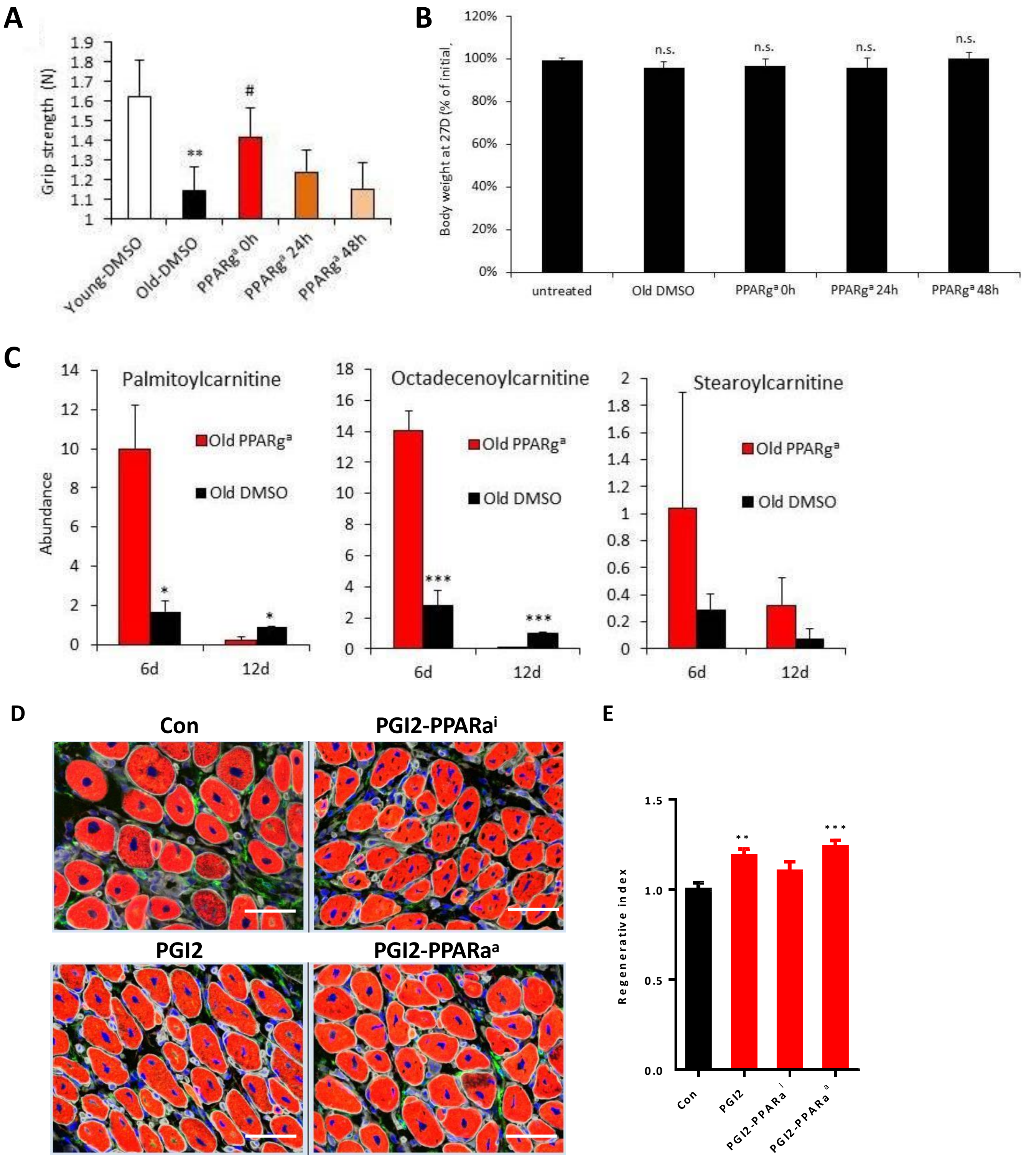

A
Grip strength (N)
1.9
1.8
1.7
1.6
1.5
1.4
1.3
1.2
1.1
1
**
#
Young-DMSO
Old-DMSO
PPARg^a 0h
PPARg^a 24h
PPARg^a 48h
B
Body weight at 27D (% of initial)
120%
100%
80%
60%
40%
20%
0%
n.s.
n.s.
n.s.
n.s.
untreated
Old DMSO
PPARg^a 0h
PPARg^a 24h
PPARg^a 48h
C
Abundance
Palmitoylcarnitine
Old PPARg^a
Old DMSO
14
12
10
8
6
4
2
0
*
*
6d
12d
Octadecenoylcarnitine
Old PPARg^a
Old DMSO
18
16
14
12
10
8
6
4
2
0
***
***
6d
12d
Stearoylcarnitine
Old PPARg^a
Old DMSO
2
1.8
1.6
1.4
1.2
1
0.8
0.6
0.4
0.2
0
6d
12d
D
Con
PGI2-PPARa^i
PGI2
PGI2-PPARa^a
E
Regenerative index
1.5
1.0
0.5
0.0
**
***
Con
PGI2
PGI2-PPARa^i
PGI2-PPARa^a

FIG. S7. PGI2's enhancement of muscle regeneration requires PPARg, not PPARa, in vivo.

(A) Assessment of grip strength in young and old mice treated with DMSO vehicle control (Young/Old DMSO), or mice treated with the PPARg agonist at 0, 24 or 48h after injury.$^{\#}$P<0.05, ** P<0.01.

(B) Total body weights (% of initial weights) of untreated old mice, old mice treated with DMSO, or old mice treated with the PPARg agonist at 0, 24 or 48h after injury, at the end of 27 days after injury.

(C) Acylcarnitine levels at 6d or 12d after injury, in old mice treated with the PPARg agonist, old mice treated with the DMSO vehicle, or young mice treated with the vehicle control . * P<0.05,** P<0.01.

(D) Immunofluorescence staining for eMHC(red) 6 days after cryoinjury of the TA muscle, followed by intramuscular injection of PGI2 at 0h and the PPARa agonist (PPARa^{a}) or PPARa inhibitor (PPARa^{i}) at 24h after injury in young mice, laminin, white; DAPI, blue. Scale bar, 50μm .

(E) Quantification of the regenerative index of muscles at 6 days after cryoinjury of young mice TA muscle, followed by intramuscular injection of PGI2 at 0h and the PPARa agonist (PPARa^{a}) or PPARa inhibitor (PPARa^{i}) at 24h after injury, relative to the vehicle control. * P<0.05, ** P<0.01,*** P<0.001.

Data were expressed as mean ± SEM. 2-tailed Student's t-test was used in (A-C) and E. N=3 biological replicates unless mentioned otherwise.

Table S1

	Species	Forward 5' to 3'	Reverse 5' to 3'
GAPDH	Human	TGGTATCGTGGAAGGACTCA	TTCAGCTCAGGGATGACCTT
PAX7	Human	GACGACGGCGAAAAGAAGG	GTAGTGGGTCCTCTCAAAGGC
PAX3	Human	CTCCACGCTCCGGATAGTTC	ATCTTGTGGCGGATGTGGTT
MYF5	Human	AATTTGGGGACGAGTTTGTG	CATGGTGGTGGACTTCCTCT
MYOD1	Human	CGGCATGATGGACTACAGCG	CAGGCAGTCTAGGCTCGAC
MYOG	Human	GGGGAAAACTACCTGCCTGTC	AGGCGCTCGATGTACTGGAT
MYHC	Human	TTCATTGGGGTCTTGGACAT	AACGTCCACTCAATGCCTTC
MYH2	Human	CTGATGCCATGGAATGACTG	CCCTATGCTTTATTTCCTTTGC
MYH3	Human	ATTGCTTCGTGGTGGACTCAA	GGCCATGTCTTCGATCCTGTC
MYH7	Human	TGCCACATCTTGATCTGCTC	CTCGGCTTCAAGGAAAATTG
MYH8	Human	TAAACACACCTGCCTGATGC	TCAGCTTTAACAGGAAAATAAACG
SkActA	Human	CGACATCAGGAAGGACCTGTATGCC	GGCCTCGTCGTACTCCTGCTTGG
NCAM1	Human	ATGGAAACTCTATTAAAGTGAACCTG	TAGACCTCATACTCAGCATTCCAGT
PPARA	Human	TCGGCGAGGATAGTTCTGGAAG	GACCACAGGATAAGTCACCGAG
PPARD	Human	GGCTTCCACTACGGTGTTCATG	CTGGCACTTGTTGCGGTTCTTC
PPARG	Human	AGCCTGCGAAAGCCTTTTGGTG	GGCTTCACATTCAGCAAACCTGG
CPT1b	Mouse	ATGTATCGCCGCAAACTGGACC	CTCTGAGAGGTGCTGTAGCAAG
ACOT1	Mouse	AAGAAGCCGTGAACTACCTGCG	TGTGATGCCCTTCAGGAAGGAG
ACOX2	Mouse	CAATGGCTTCCTGCGACTGAAC	AAGCCTCTGGTAGGTGCCATCT
CD36	Mouse	GGACATTGAGATTCTTTTCCTCTG	GCAAAGGCATTGGCTGGAAGAAC
ACAD1	Mouse	GGCGATTTCTGCCTGTGAGTTC	GCTGTCCACAAAAGCTCTGGTG
ACADSB	Mouse	TGGAAGCCACACGGTTGCTAAC	CATCCACTCGATGCACTTGCTTG
GAPDH	Mouse	CATCACTGCCACCCAGAAGACTG	ATGCCAGTGAGCTTCCCGTTCAG

Table S1. Primers used for qRT-PCR of mouse and human genes.

Table S2

	Species	Forward 5' to 3'	Reverse 5' to 3'
B2M	Human	CACTGAAAAAGATGAGTATGCC	AACATTCCCTGACAATCCC
ND1	Human	ACGCCATAAAACTCTTCACCAAAG	GGGTTCATAGTAGAAGAGCGATGG
ND4	Human	ACCTTGGCTATCATCACCCGAT	AGTGCGATGAGTAGGGGAAGG
ND5	Human	AGTTACAATCGGCATCAACCAA	CCCGGAGCACATAAATAGTATGG
ND6	Human	TGGGGTTAGCGATGGAGGTAGG	AATAGGATCCTCCCGAATCAAC

Table S2. Primers used for quantitative PCR of mitochondrial DNA (mitochondrial ND, NADH dehydrogenase genes) and nuclear DNA (B2M, beta-2-microglobulin).
